# Supplementary material for: Using Cognitive Load Theory to Improve Teaching in the Clinical Workplace
Source: MedEdPORTAL. 2020 Oct 2;16:10983. doi: 10.15766/mep_2374-8265.10983 (PMC7549387; doi:10.15766/mep_2374-8265.10983)
Supplement: Supplementary file 1 — Large-Group CLT Overview.pptxActivity 1 Small-Group Worked Example.docxActivity 2 Individual Activity Design.docxWorkshop Participant Evaluations.docxFollow-Up Survey.docxFacilitator Guide.docx [file mep_2374-8265.10983-s001.zip › A. Large-Group CLT Overview.pptx]

## Slide 1
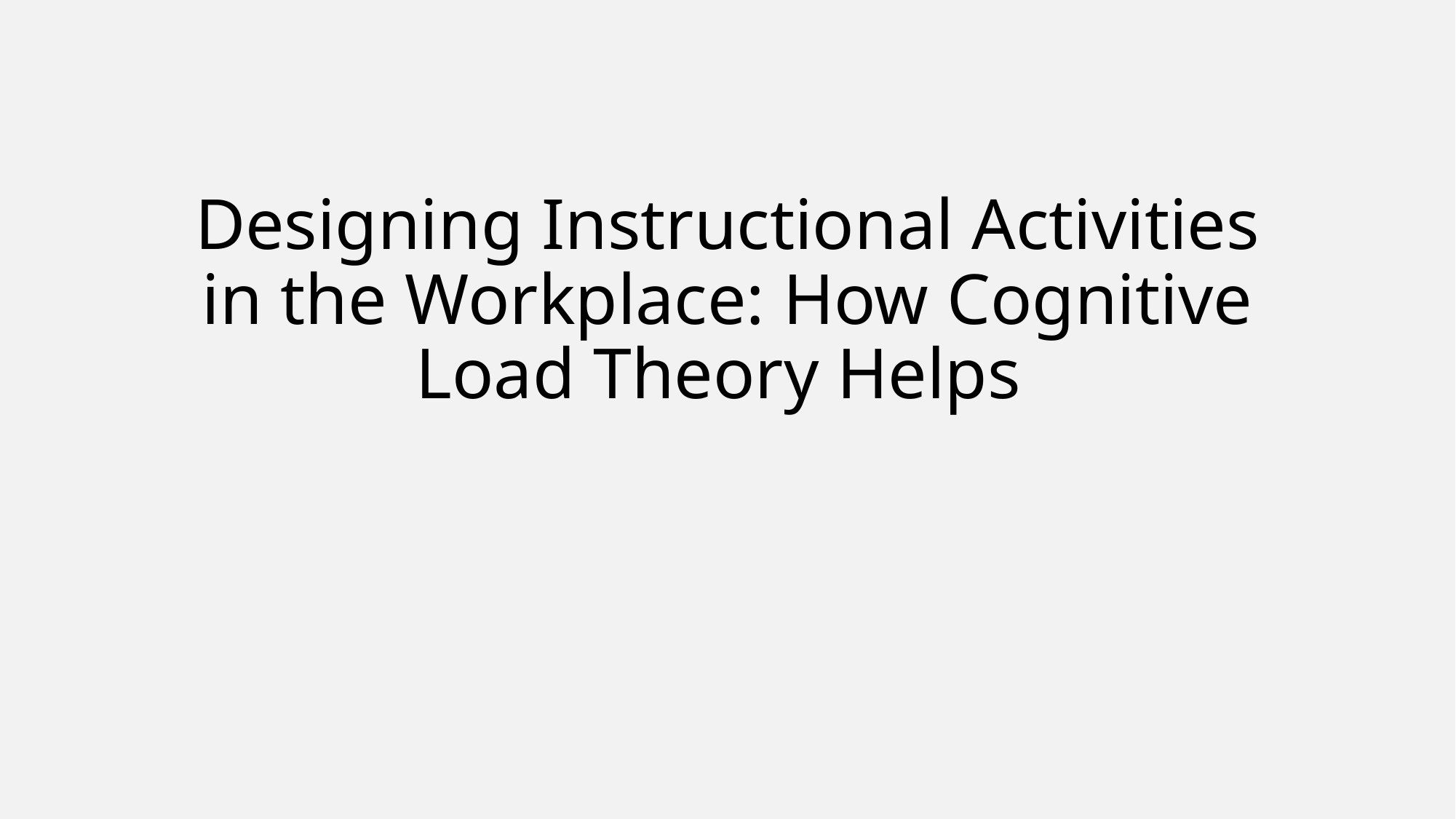

# Designing Instructional Activities in the Workplace: How Cognitive Load Theory Helps

## Slide 2
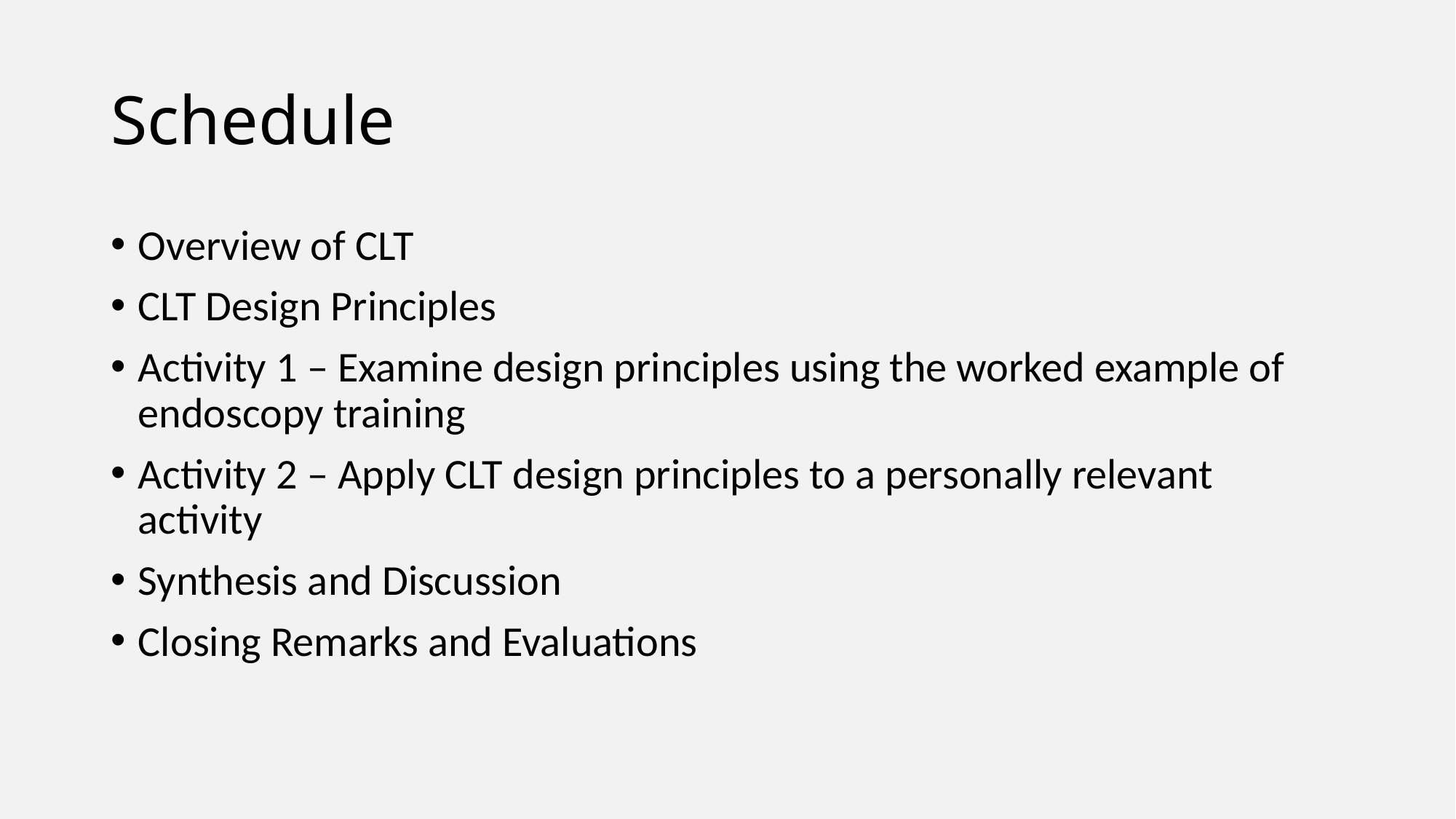

# Schedule
Overview of CLT
CLT Design Principles
Activity 1 – Examine design principles using the worked example of endoscopy training
Activity 2 – Apply CLT design principles to a personally relevant activity
Synthesis and Discussion
Closing Remarks and Evaluations

## Slide 3
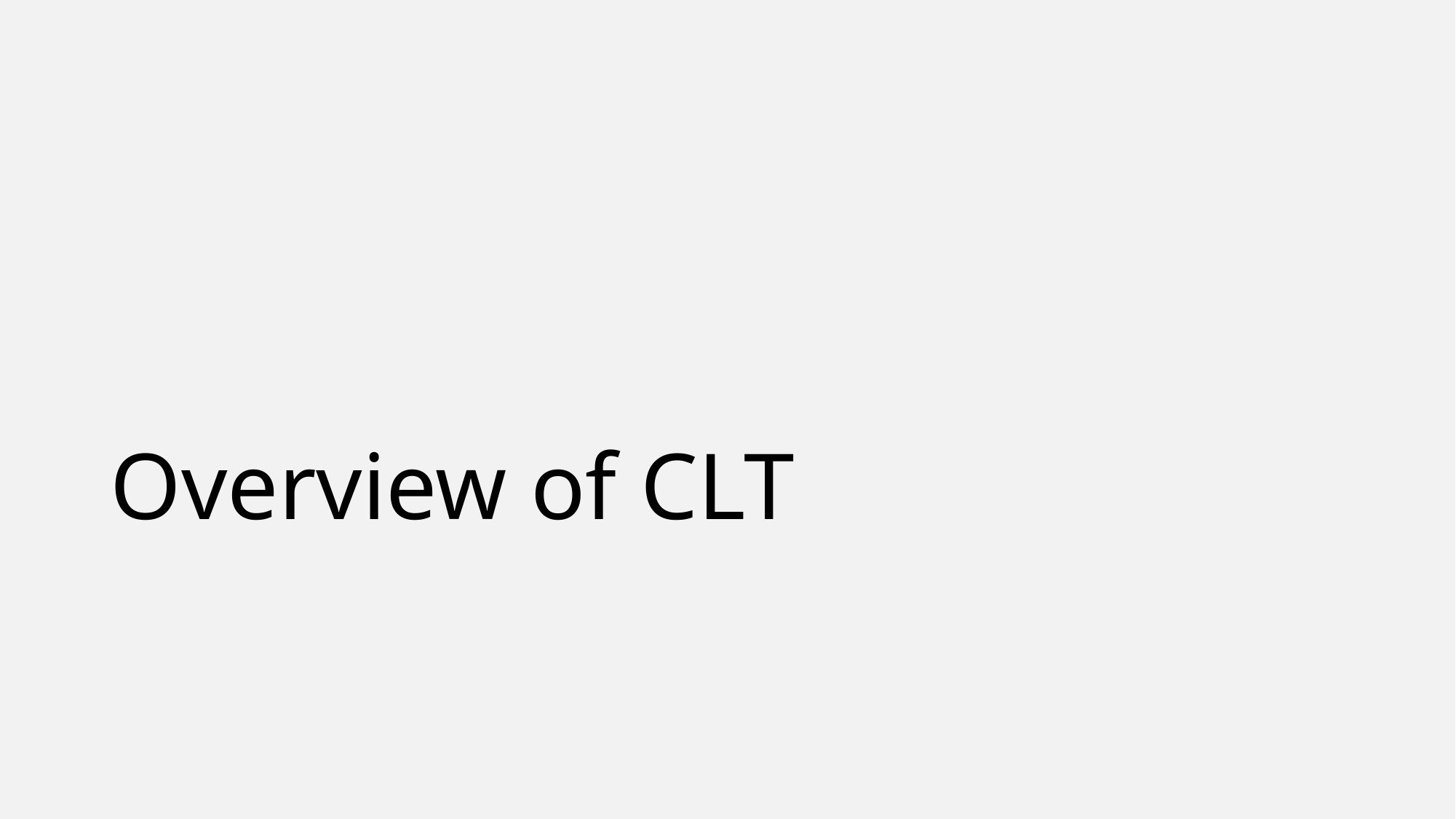

# Overview of CLT

## Slide 4
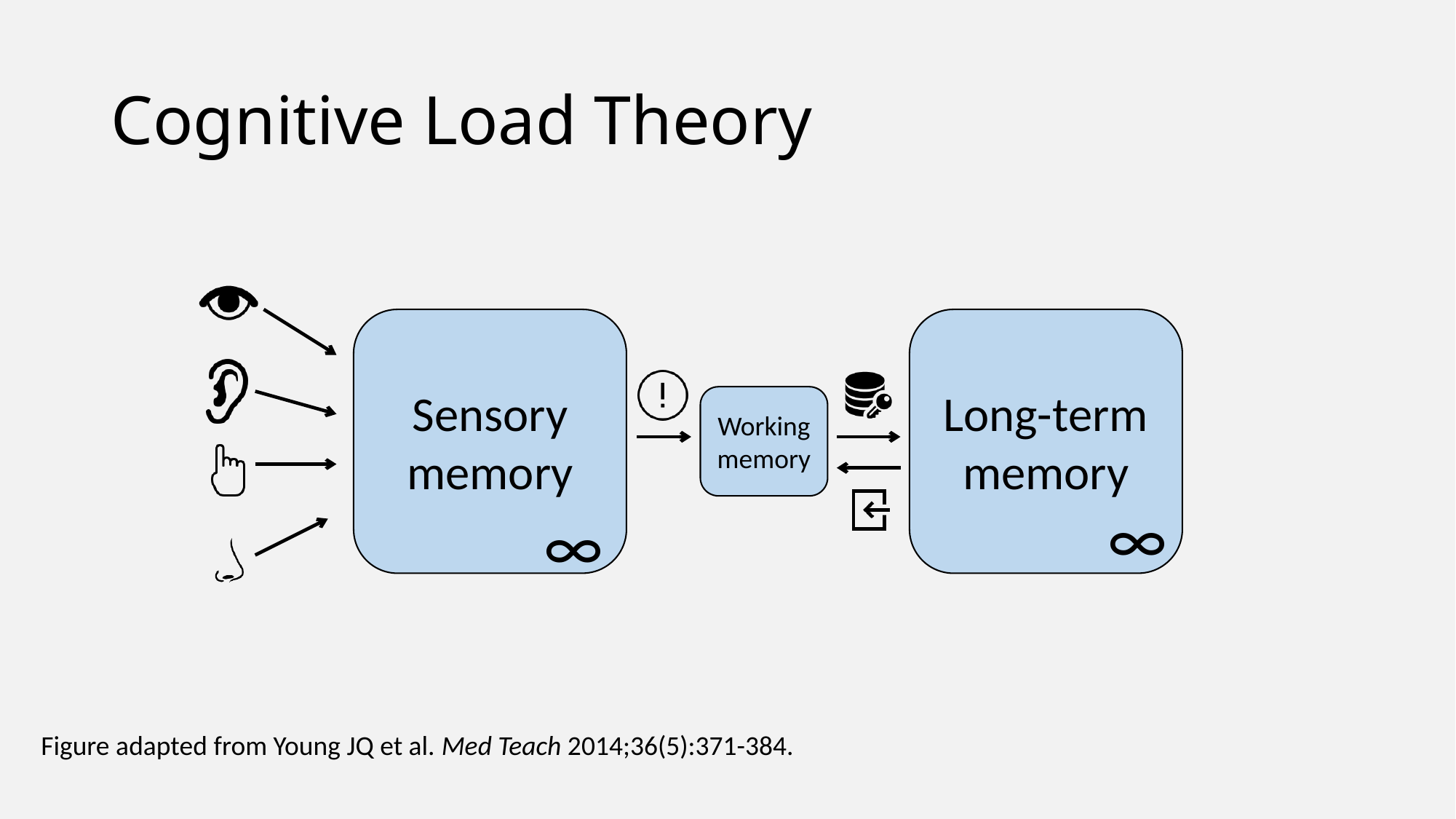

# Cognitive Load Theory
Sensory memory
Long-term memory
Working memory
∞
∞
Figure adapted from Young JQ et al. Med Teach 2014;36(5):371-384.

## Slide 5
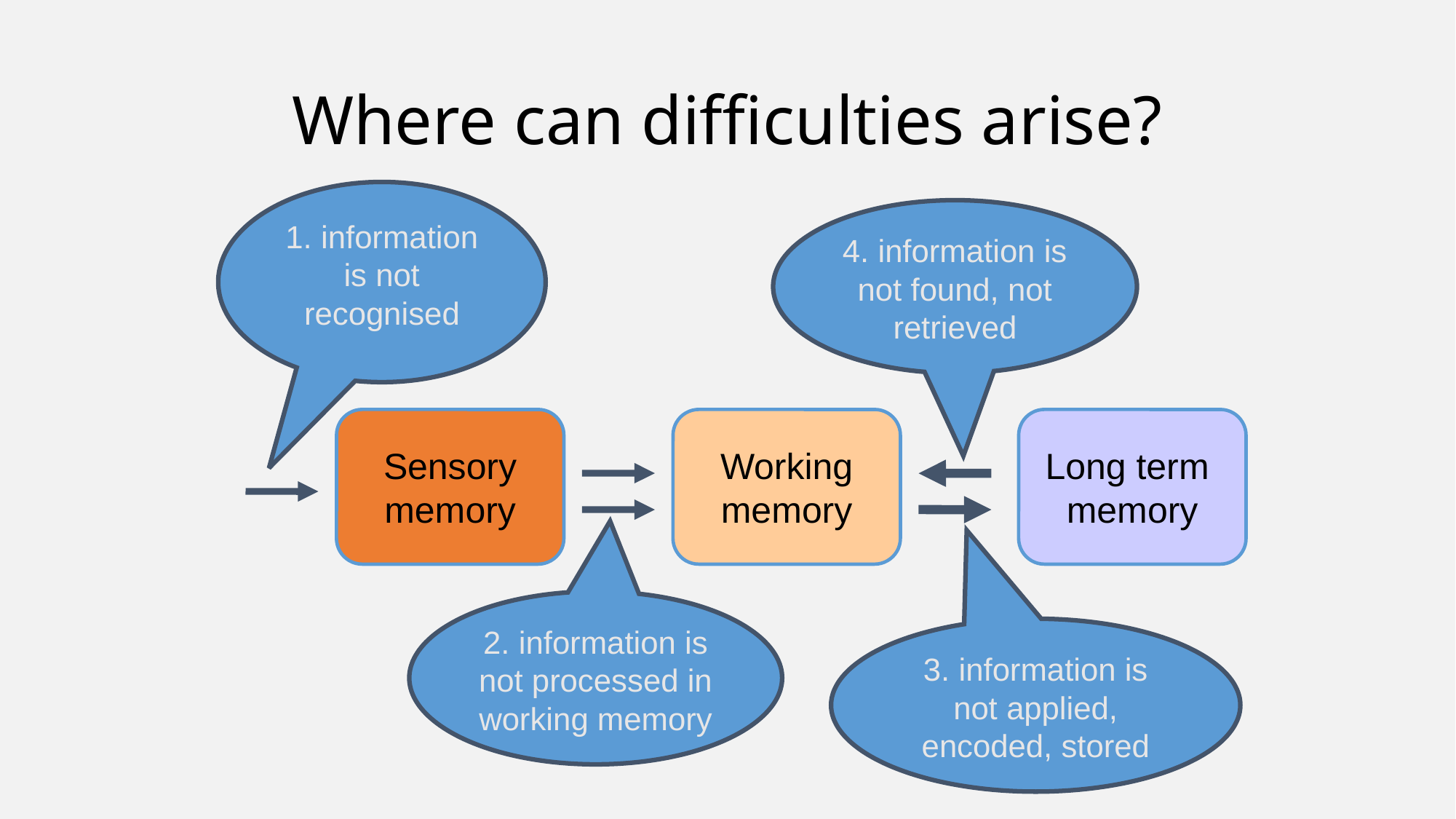

# Where can difficulties arise?
1. information is not recognised
4. information is not found, not retrieved
Sensory
memory
Working
memory
Long term
memory
2. information is not processed in working memory
3. information is not applied, encoded, stored

## Slide 6
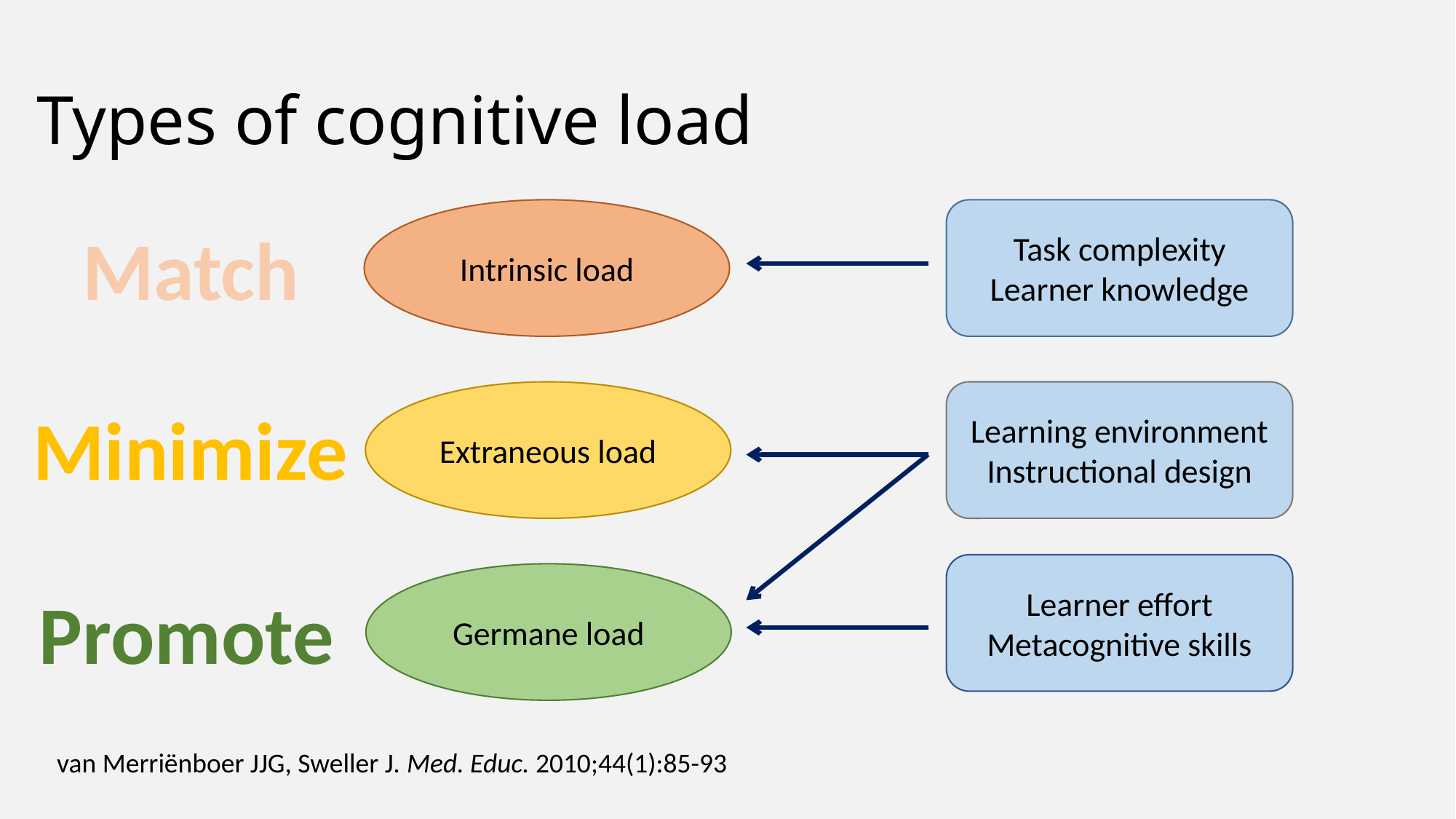

# Types of cognitive load
Intrinsic load
Task complexity Learner knowledge
Match
Extraneous load
Learning environment
Instructional design
Minimize
Learner effort Metacognitive skills
Germane load
Promote
van Merriënboer JJG, Sweller J. Med. Educ. 2010;44(1):85-93

## Slide 7
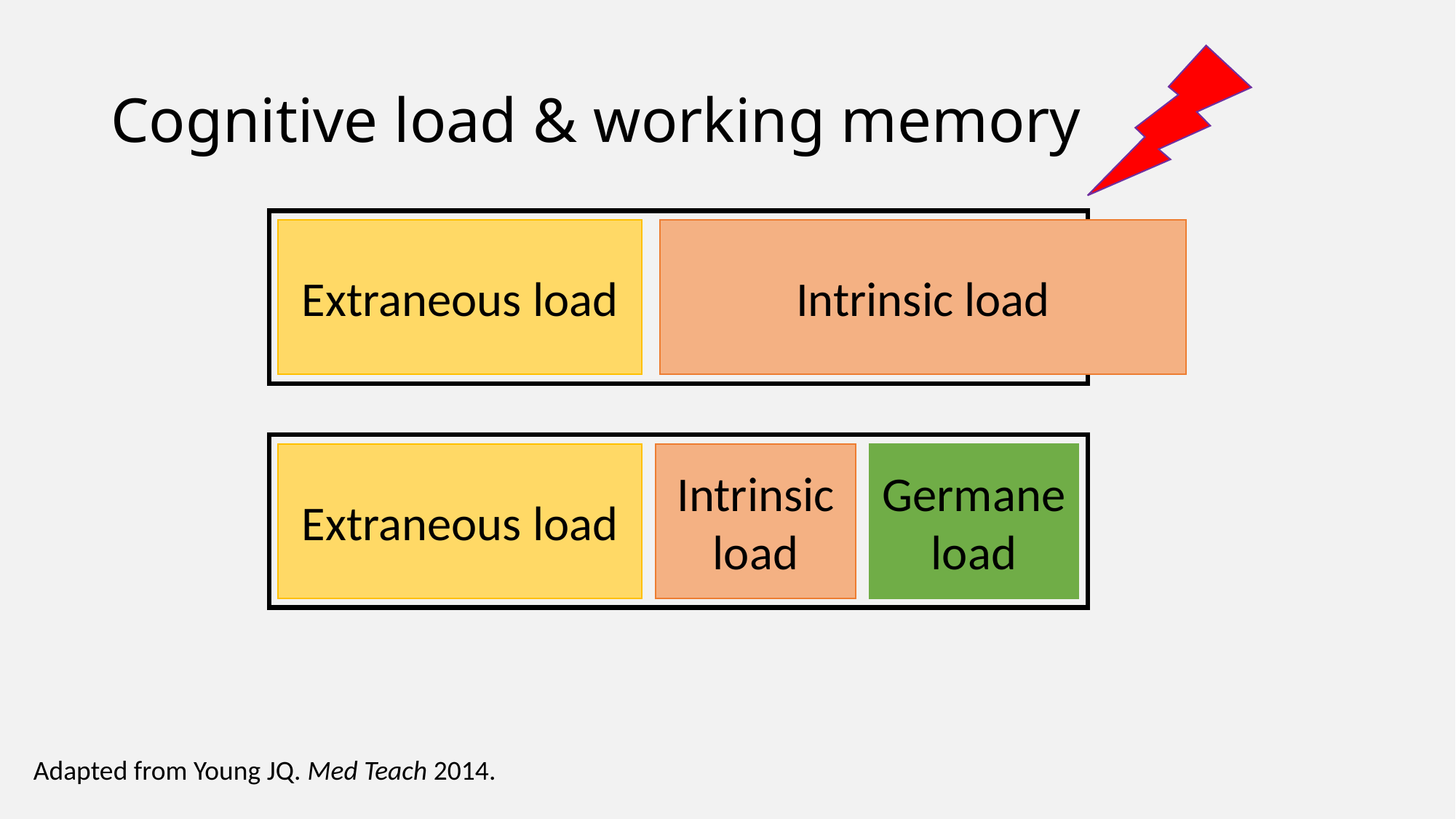

# Cognitive load & working memory
Extraneous load
Intrinsic load
Extraneous load
Intrinsic load
Germane load
Adapted from Young JQ. Med Teach 2014.

## Slide 8
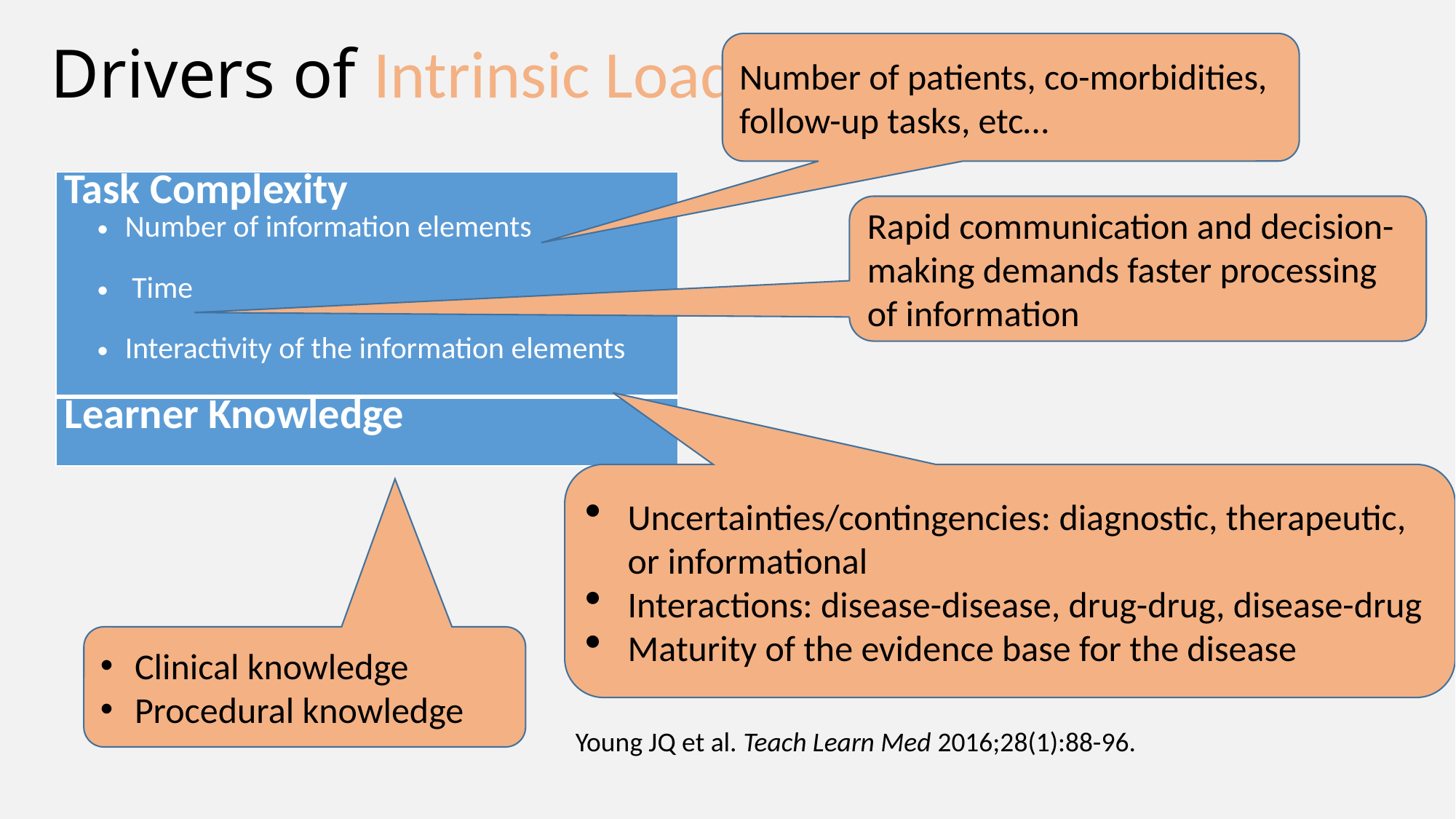

# Drivers of Intrinsic Load
Number of patients, co-morbidities, follow-up tasks, etc…
| Task Complexity Number of information elements  Time Interactivity of the information elements |
| --- |
| Learner Knowledge |
Rapid communication and decision-making demands faster processing of information
Uncertainties/contingencies: diagnostic, therapeutic, or informational
Interactions: disease-disease, drug-drug, disease-drug
Maturity of the evidence base for the disease
Clinical knowledge
Procedural knowledge
Young JQ et al. Teach Learn Med 2016;28(1):88-96.

## Slide 9
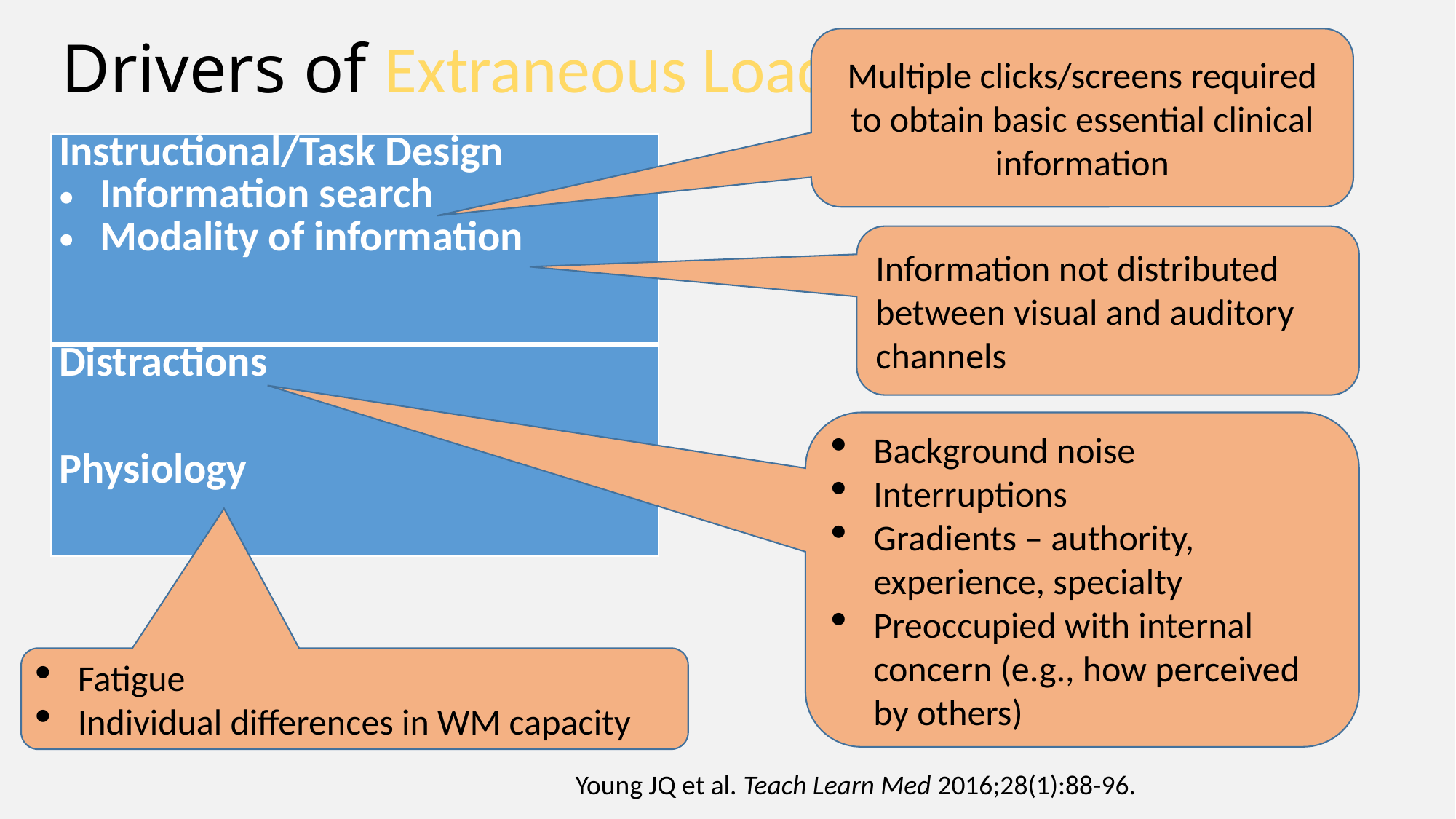

# Drivers of Extraneous Load
Multiple clicks/screens required to obtain basic essential clinical information
| Instructional/Task Design Information search Modality of information |
| --- |
| Distractions |
| Physiology |
Information not distributed between visual and auditory channels
Background noise
Interruptions
Gradients – authority, experience, specialty
Preoccupied with internal concern (e.g., how perceived by others)
Fatigue
Individual differences in WM capacity
Young JQ et al. Teach Learn Med 2016;28(1):88-96.

## Slide 10
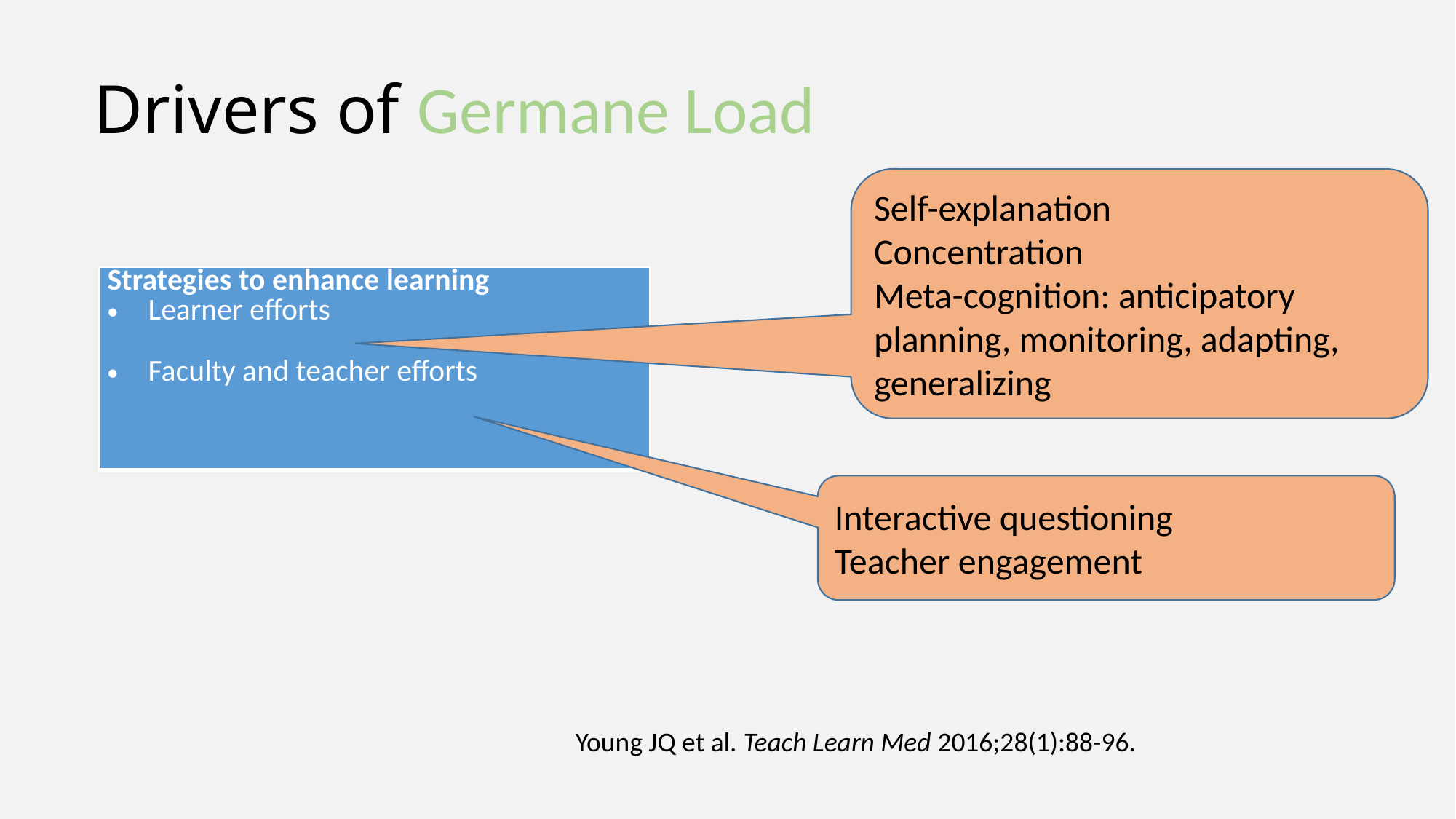

# Drivers of Germane Load
Self-explanation
Concentration
Meta-cognition: anticipatory planning, monitoring, adapting, generalizing
| Strategies to enhance learning Learner efforts Faculty and teacher efforts |
| --- |
Interactive questioning
Teacher engagement
Young JQ et al. Teach Learn Med 2016;28(1):88-96.

## Slide 11
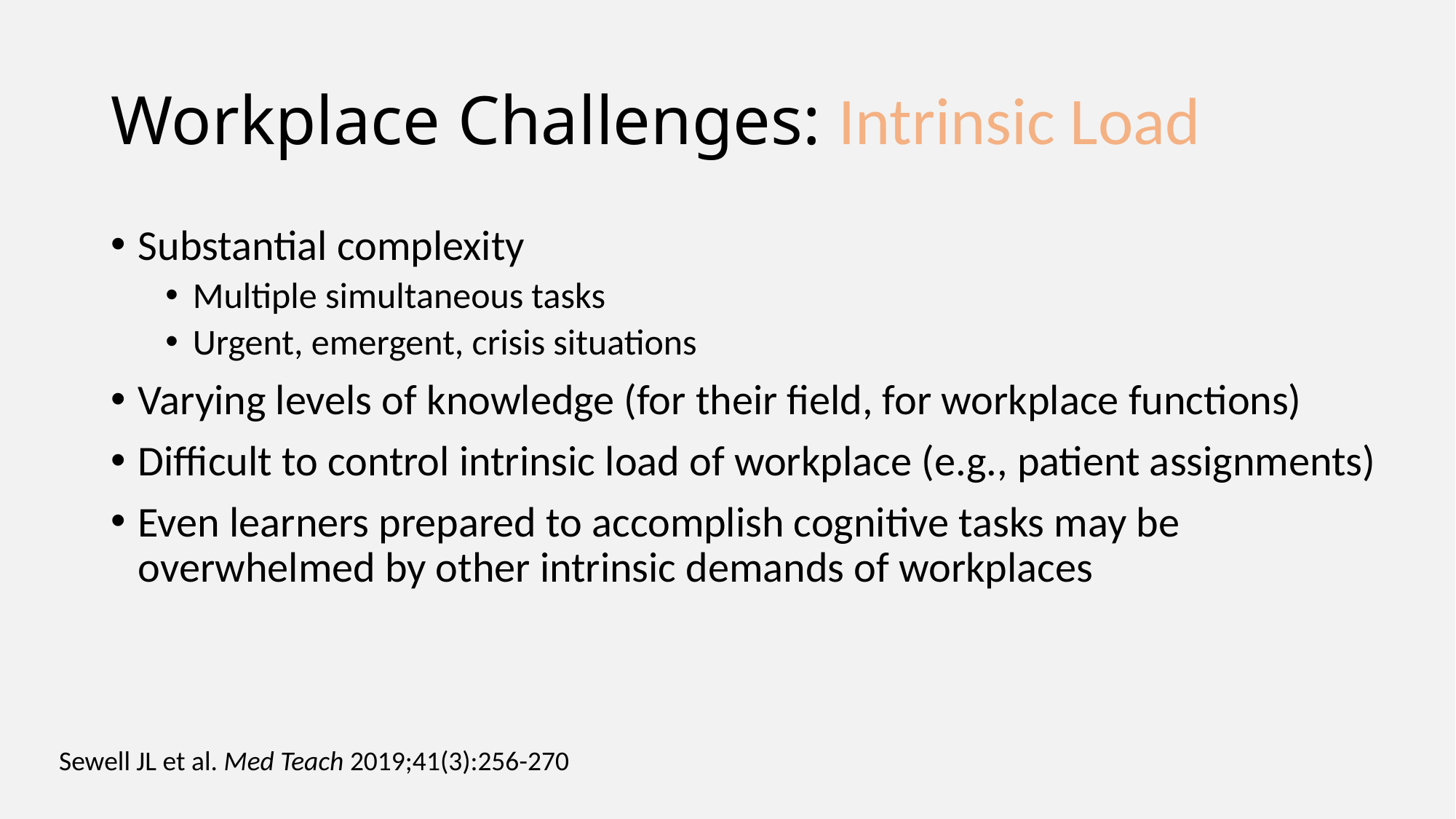

# Workplace Challenges: Intrinsic Load
Substantial complexity
Multiple simultaneous tasks
Urgent, emergent, crisis situations
Varying levels of knowledge (for their field, for workplace functions)
Difficult to control intrinsic load of workplace (e.g., patient assignments)
Even learners prepared to accomplish cognitive tasks may be overwhelmed by other intrinsic demands of workplaces
Sewell JL et al. Med Teach 2019;41(3):256-270

## Slide 12
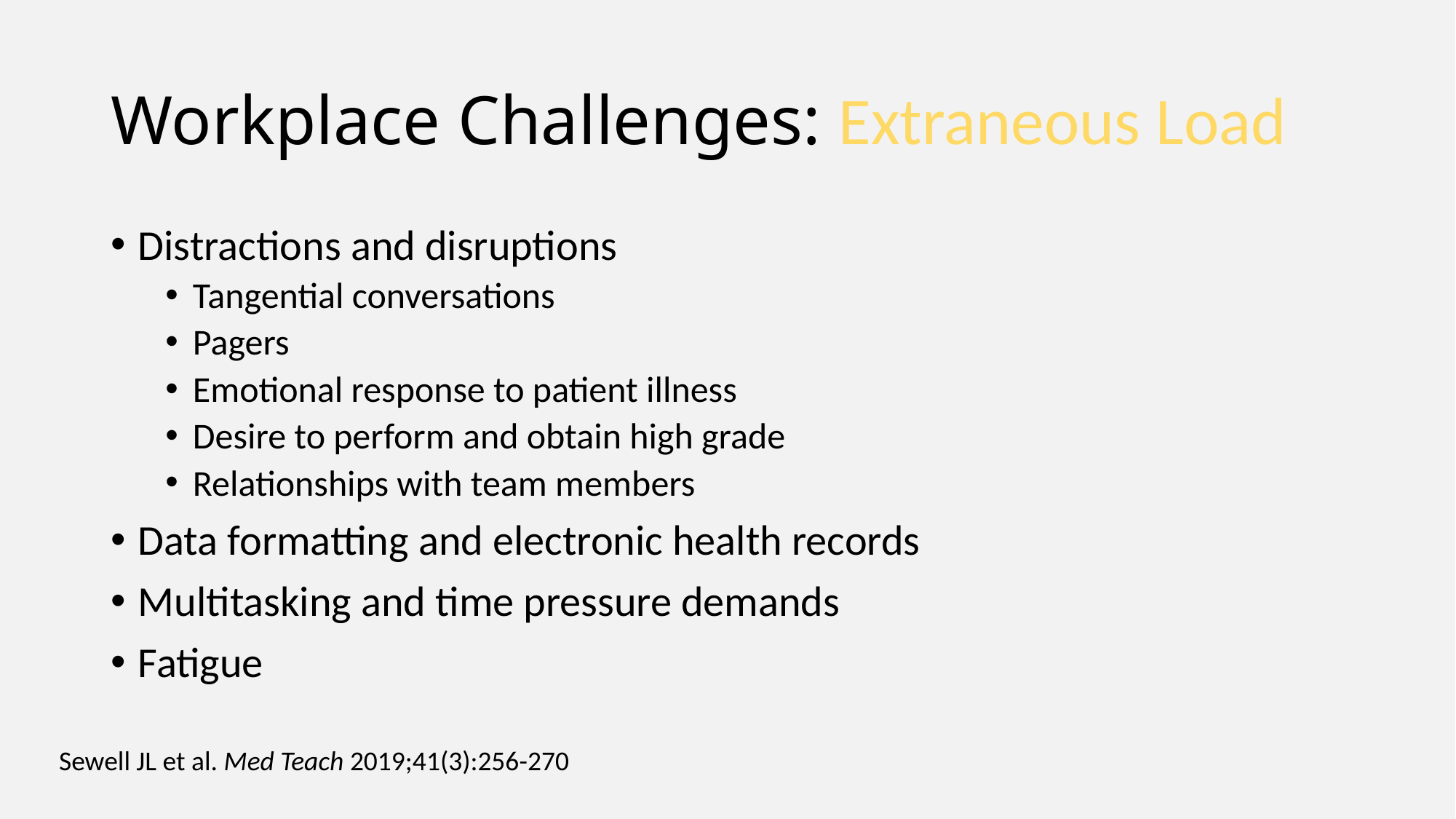

# Workplace Challenges: Extraneous Load
Distractions and disruptions
Tangential conversations
Pagers
Emotional response to patient illness
Desire to perform and obtain high grade
Relationships with team members
Data formatting and electronic health records
Multitasking and time pressure demands
Fatigue
Sewell JL et al. Med Teach 2019;41(3):256-270

## Slide 13
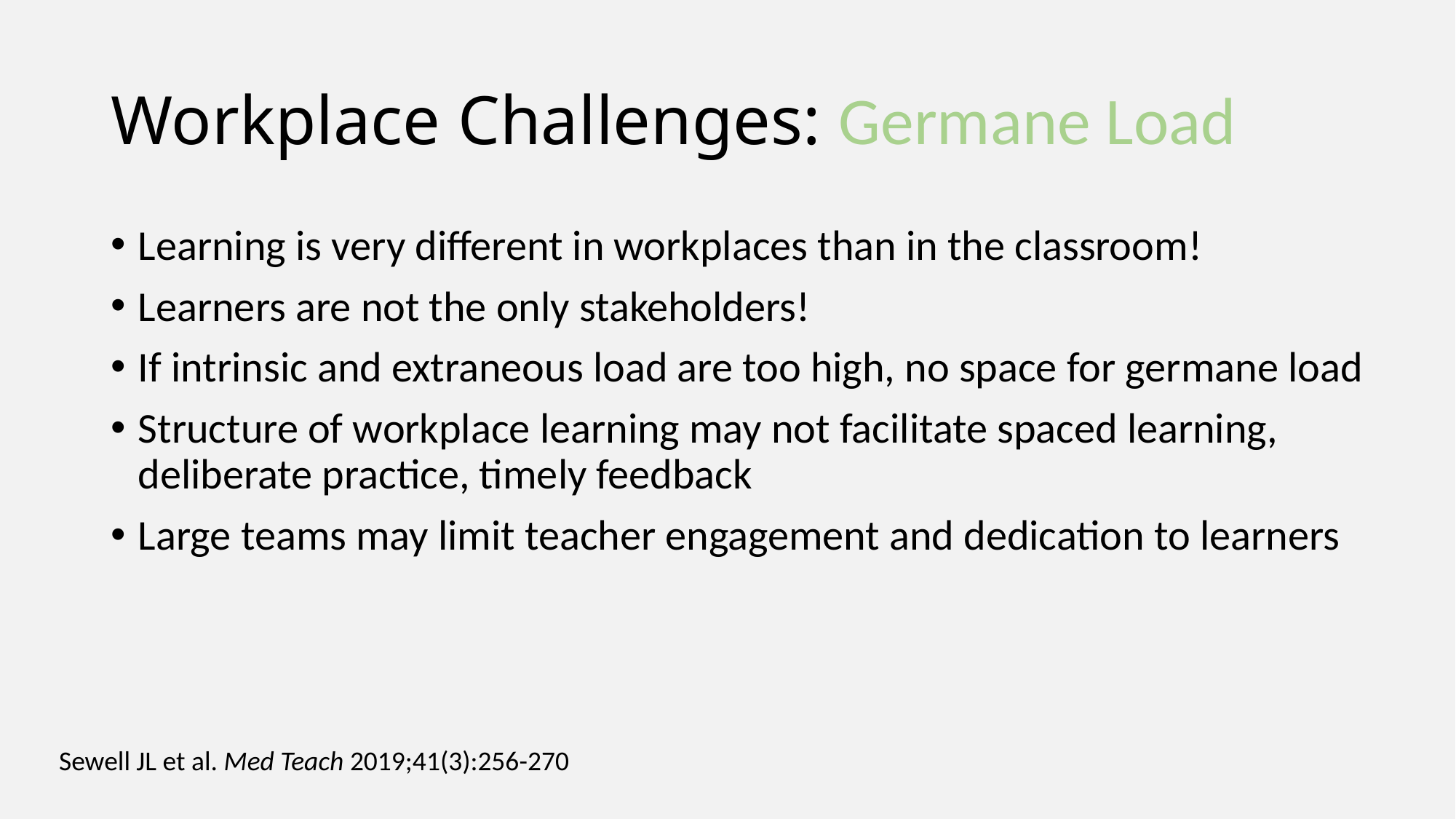

# Workplace Challenges: Germane Load
Learning is very different in workplaces than in the classroom!
Learners are not the only stakeholders!
If intrinsic and extraneous load are too high, no space for germane load
Structure of workplace learning may not facilitate spaced learning, deliberate practice, timely feedback
Large teams may limit teacher engagement and dedication to learners
Sewell JL et al. Med Teach 2019;41(3):256-270

## Slide 14
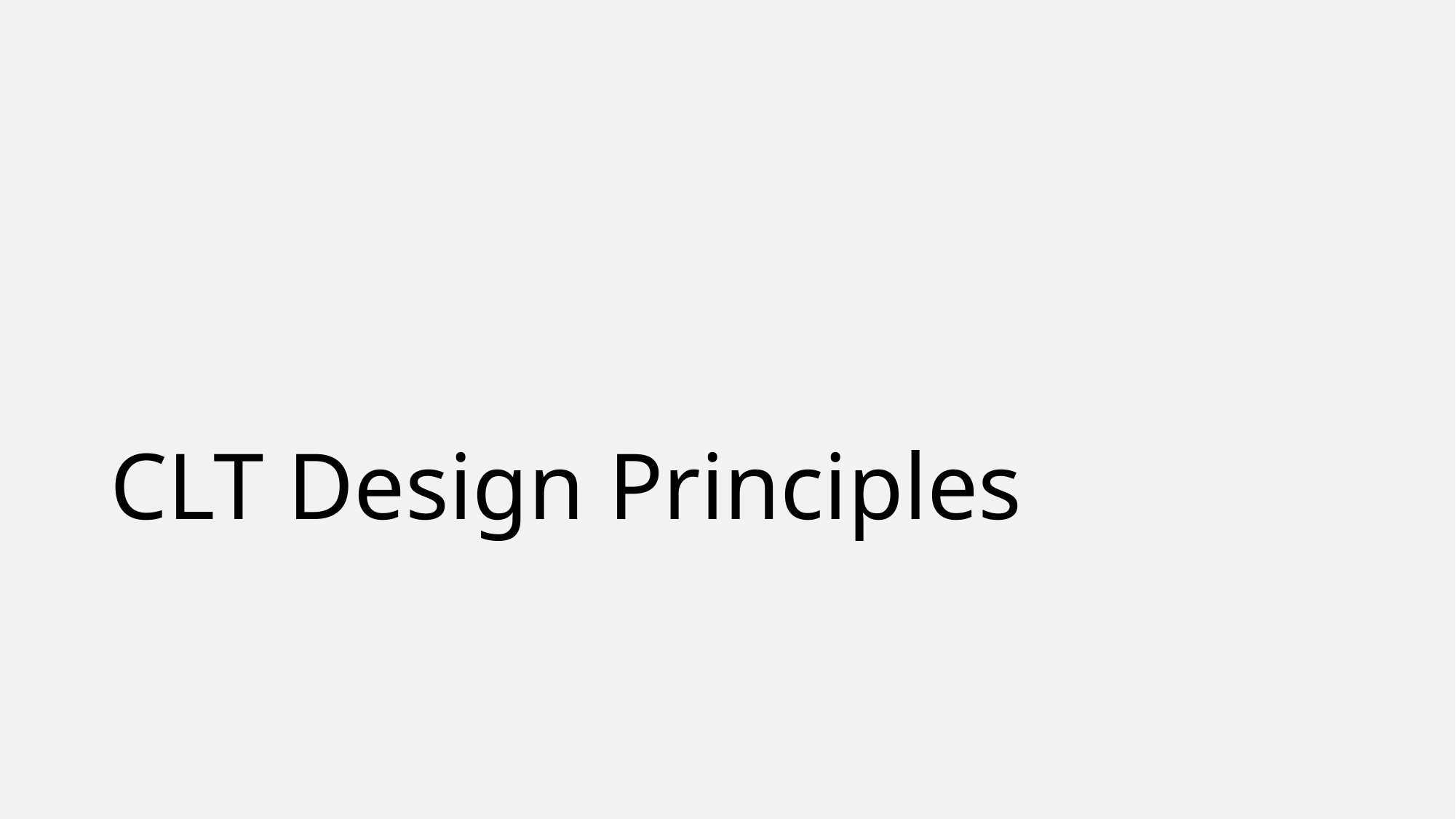

# CLT Design Principles

## Slide 15
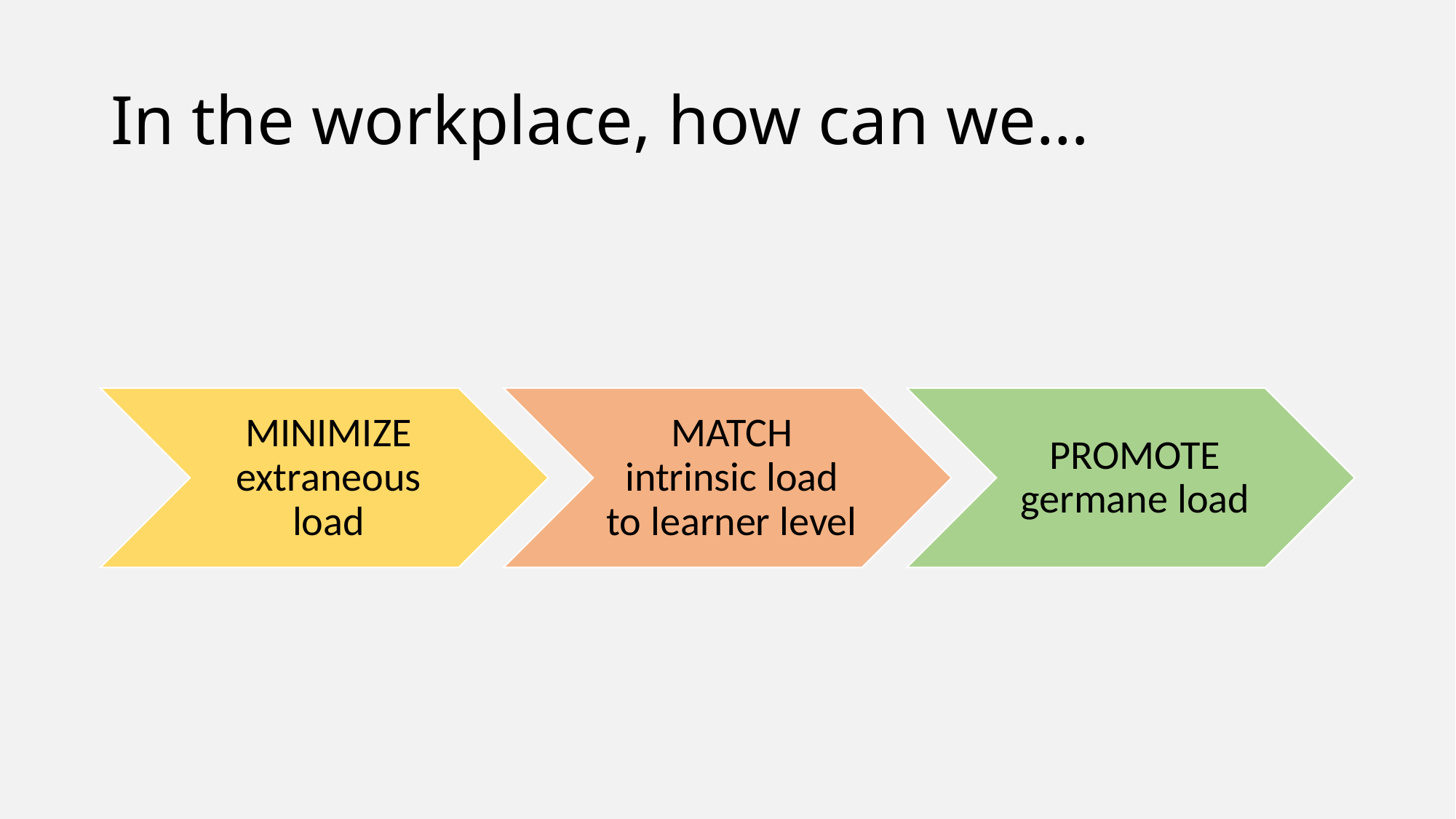

# In the workplace, how can we…

## Slide 16
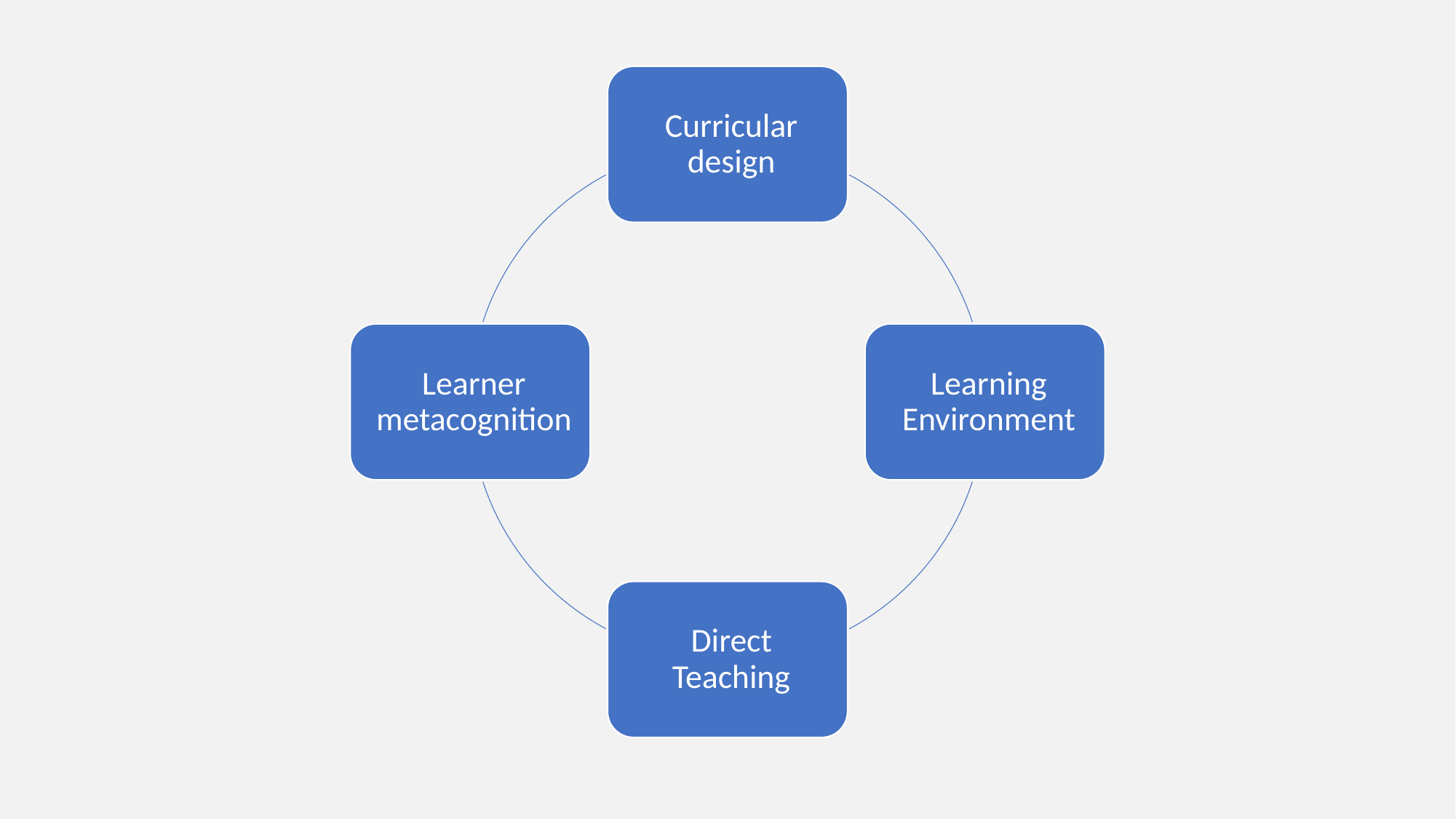

## Slide 17
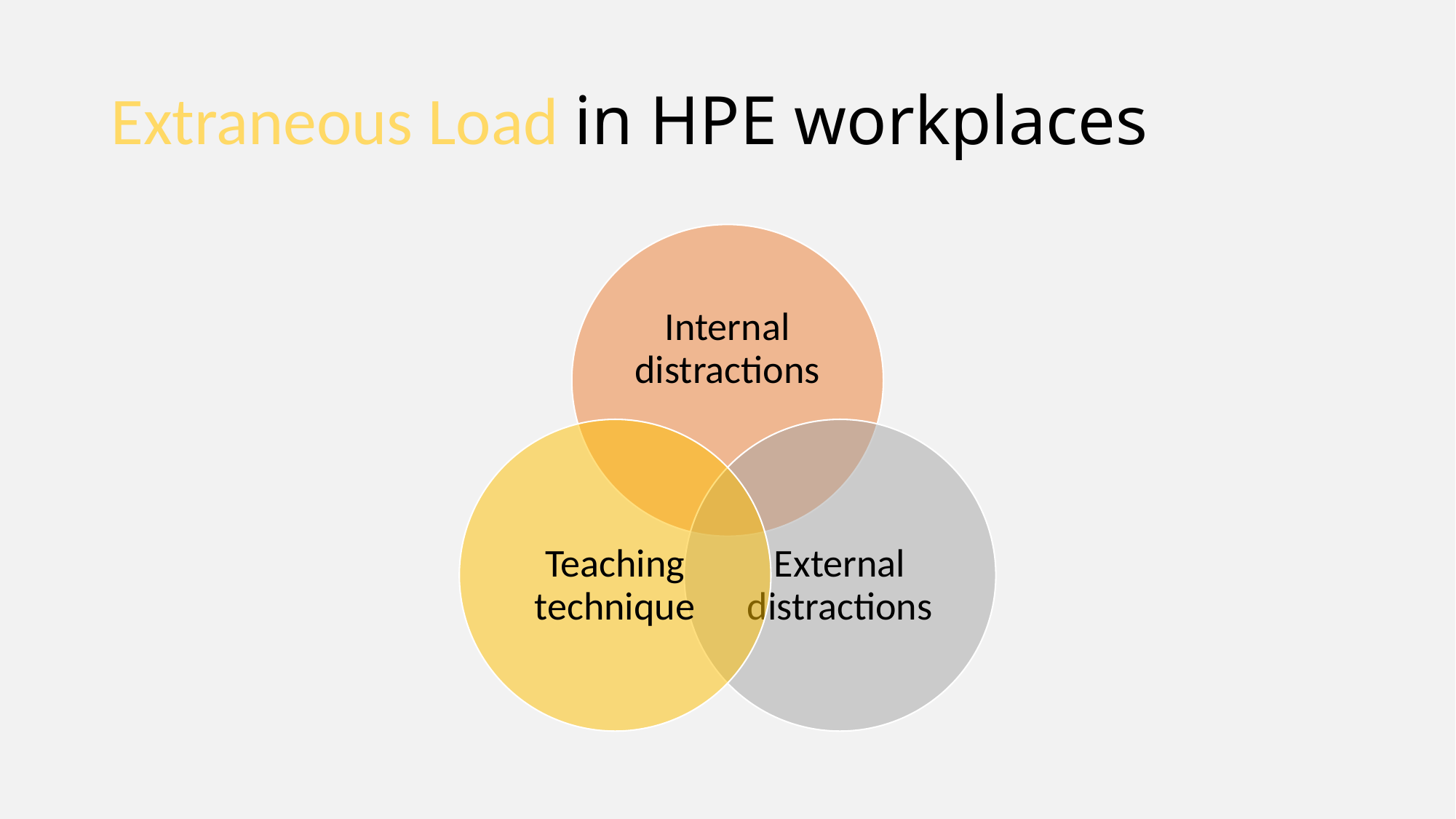

# Extraneous Load in HPE workplaces

## Slide 18
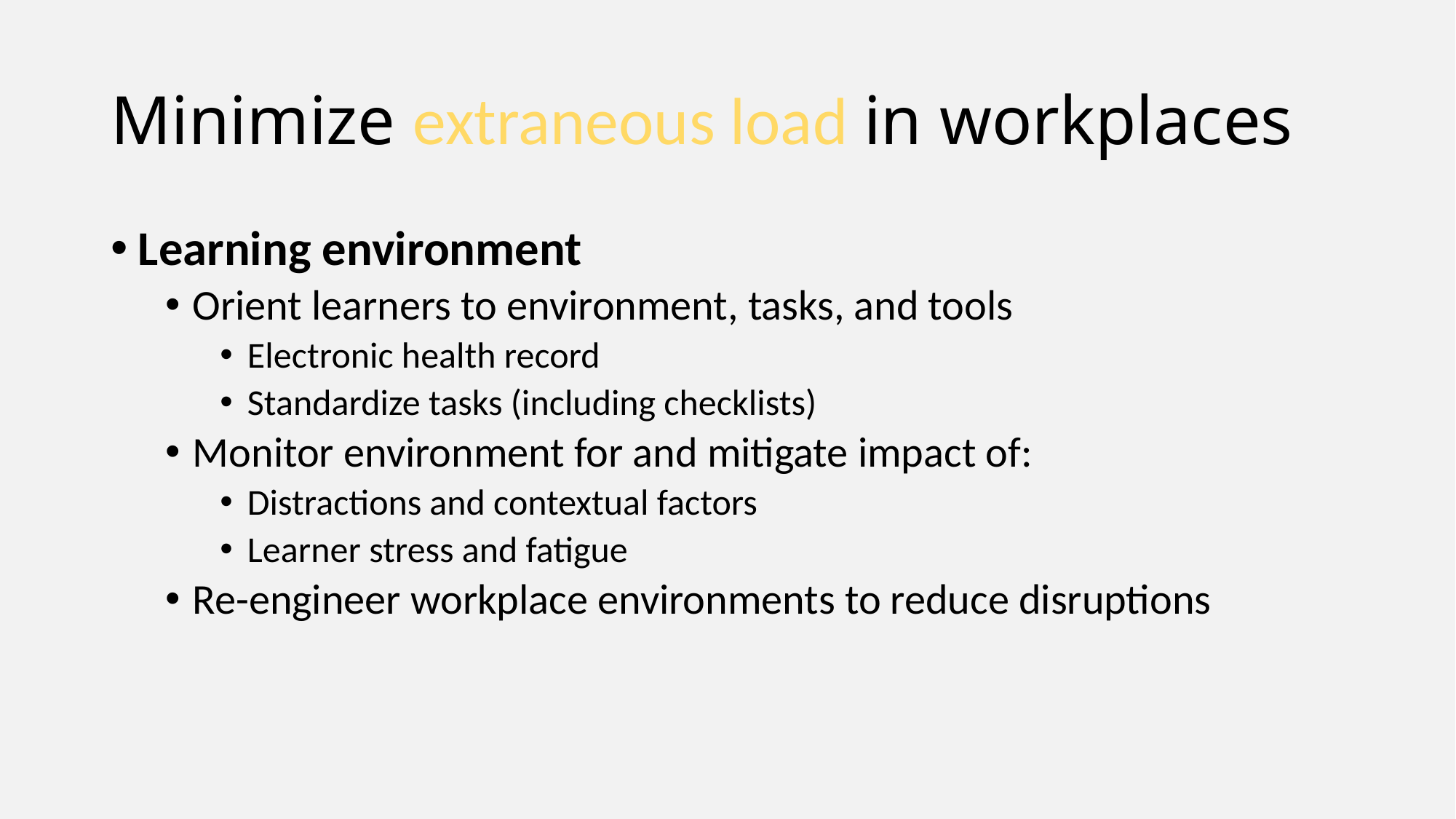

# Minimize extraneous load in workplaces
Learning environment
Orient learners to environment, tasks, and tools
Electronic health record
Standardize tasks (including checklists)
Monitor environment for and mitigate impact of:
Distractions and contextual factors
Learner stress and fatigue
Re-engineer workplace environments to reduce disruptions

## Slide 19
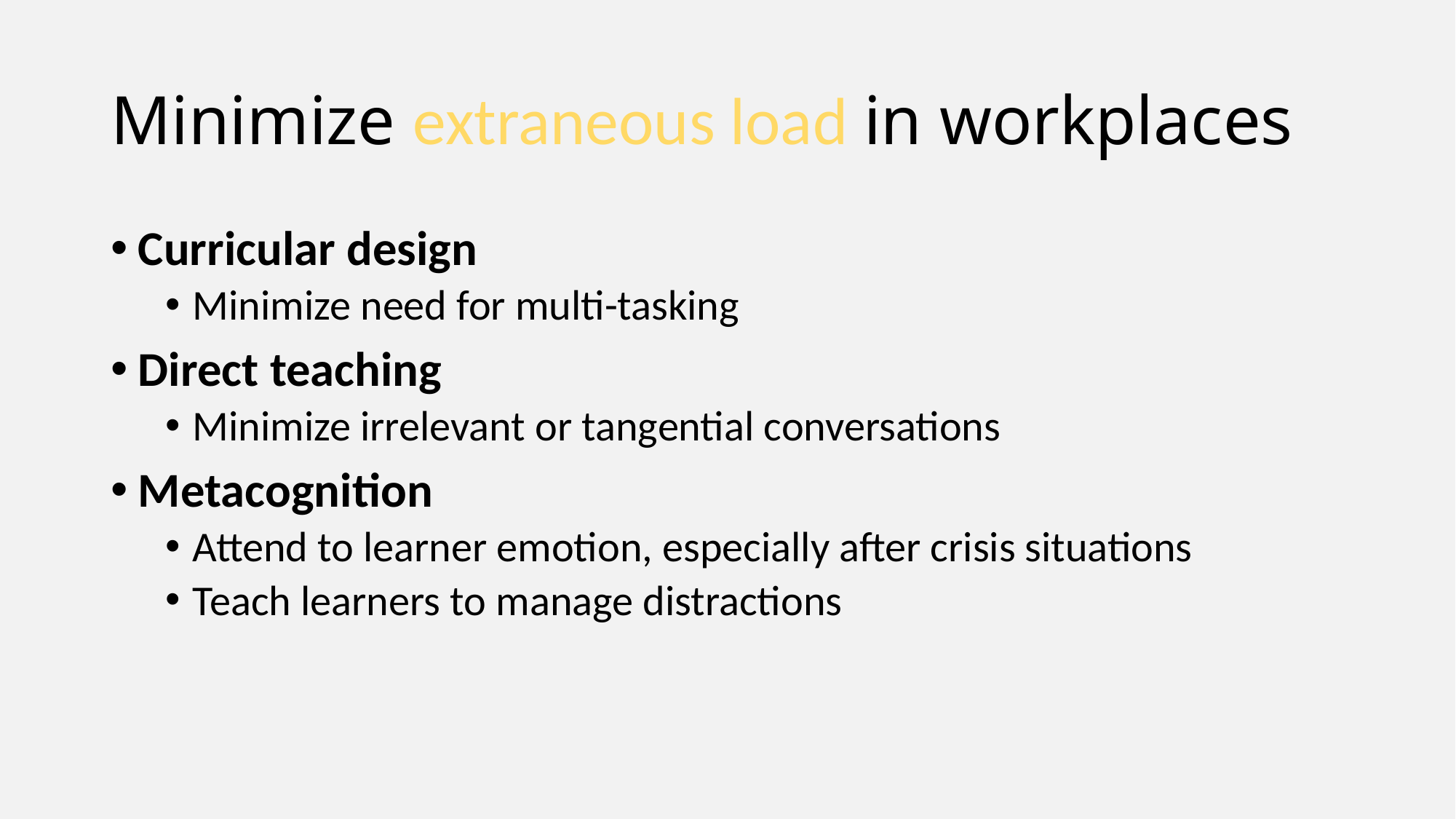

# Minimize extraneous load in workplaces
Curricular design
Minimize need for multi-tasking
Direct teaching
Minimize irrelevant or tangential conversations
Metacognition
Attend to learner emotion, especially after crisis situations
Teach learners to manage distractions

## Slide 20
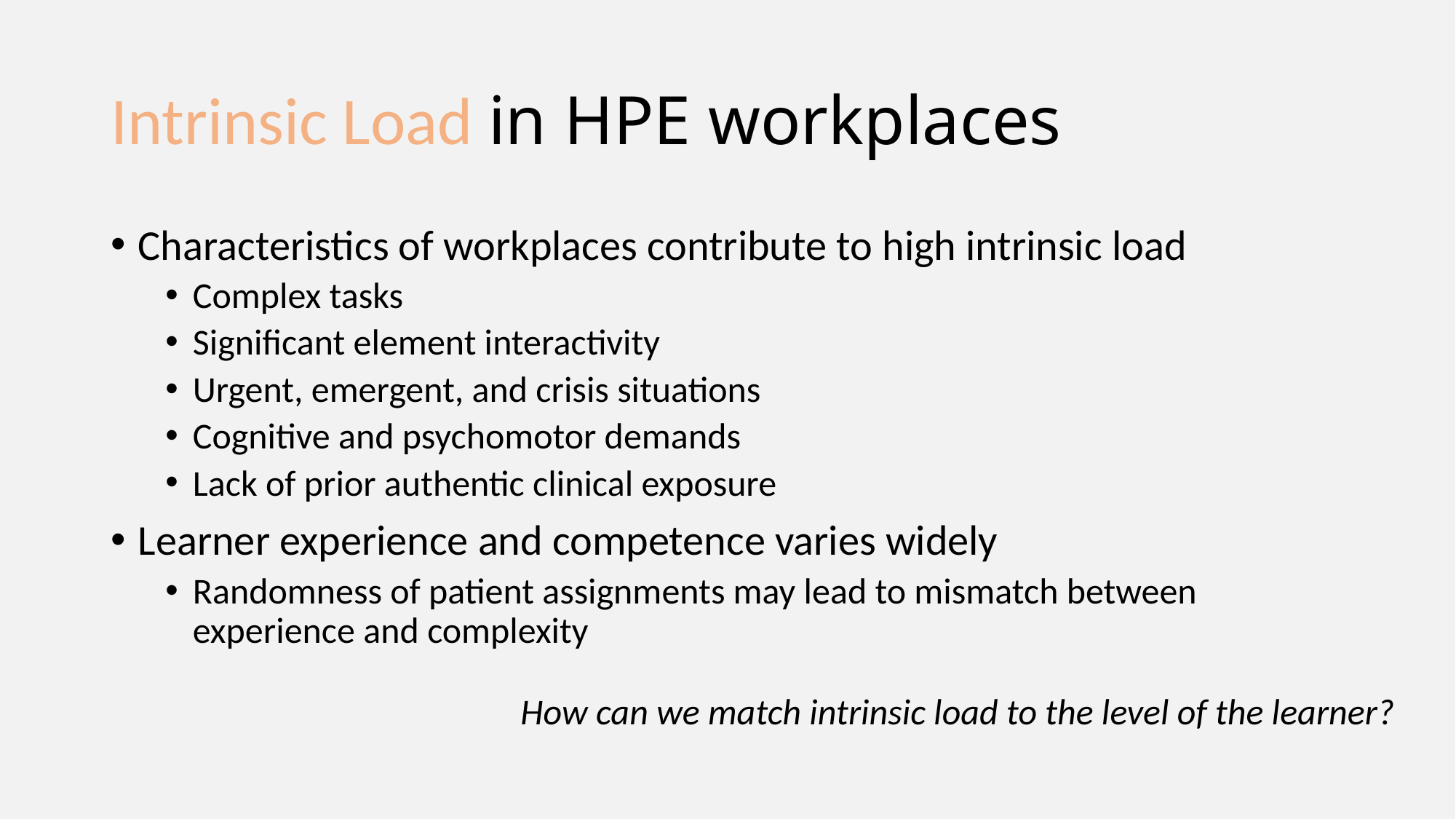

# Intrinsic Load in HPE workplaces
Characteristics of workplaces contribute to high intrinsic load
Complex tasks
Significant element interactivity
Urgent, emergent, and crisis situations
Cognitive and psychomotor demands
Lack of prior authentic clinical exposure
Learner experience and competence varies widely
Randomness of patient assignments may lead to mismatch between experience and complexity
How can we match intrinsic load to the level of the learner?

## Slide 21
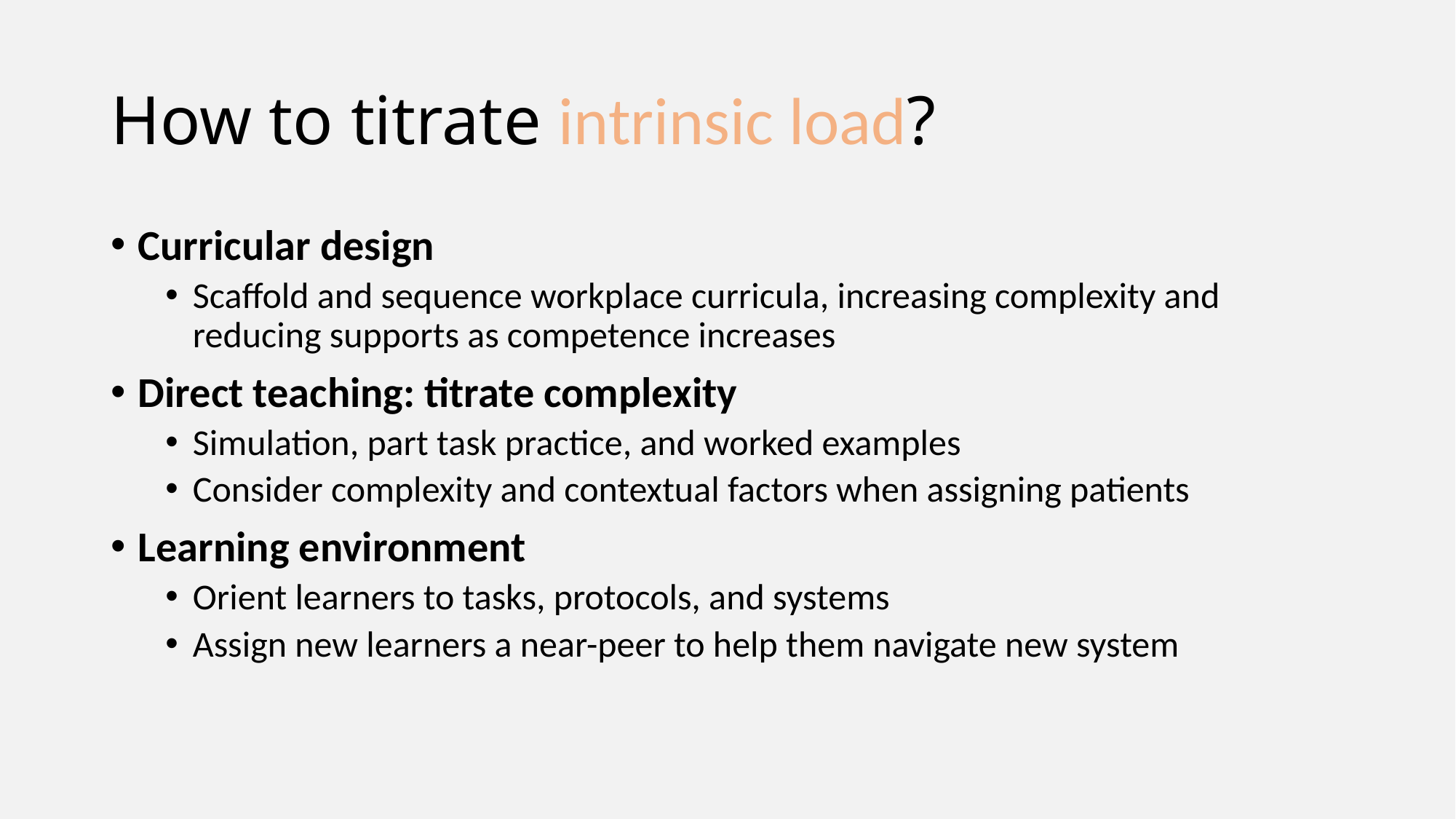

# How to titrate intrinsic load?
Curricular design
Scaffold and sequence workplace curricula, increasing complexity and reducing supports as competence increases
Direct teaching: titrate complexity
Simulation, part task practice, and worked examples
Consider complexity and contextual factors when assigning patients
Learning environment
Orient learners to tasks, protocols, and systems
Assign new learners a near-peer to help them navigate new system

## Slide 22
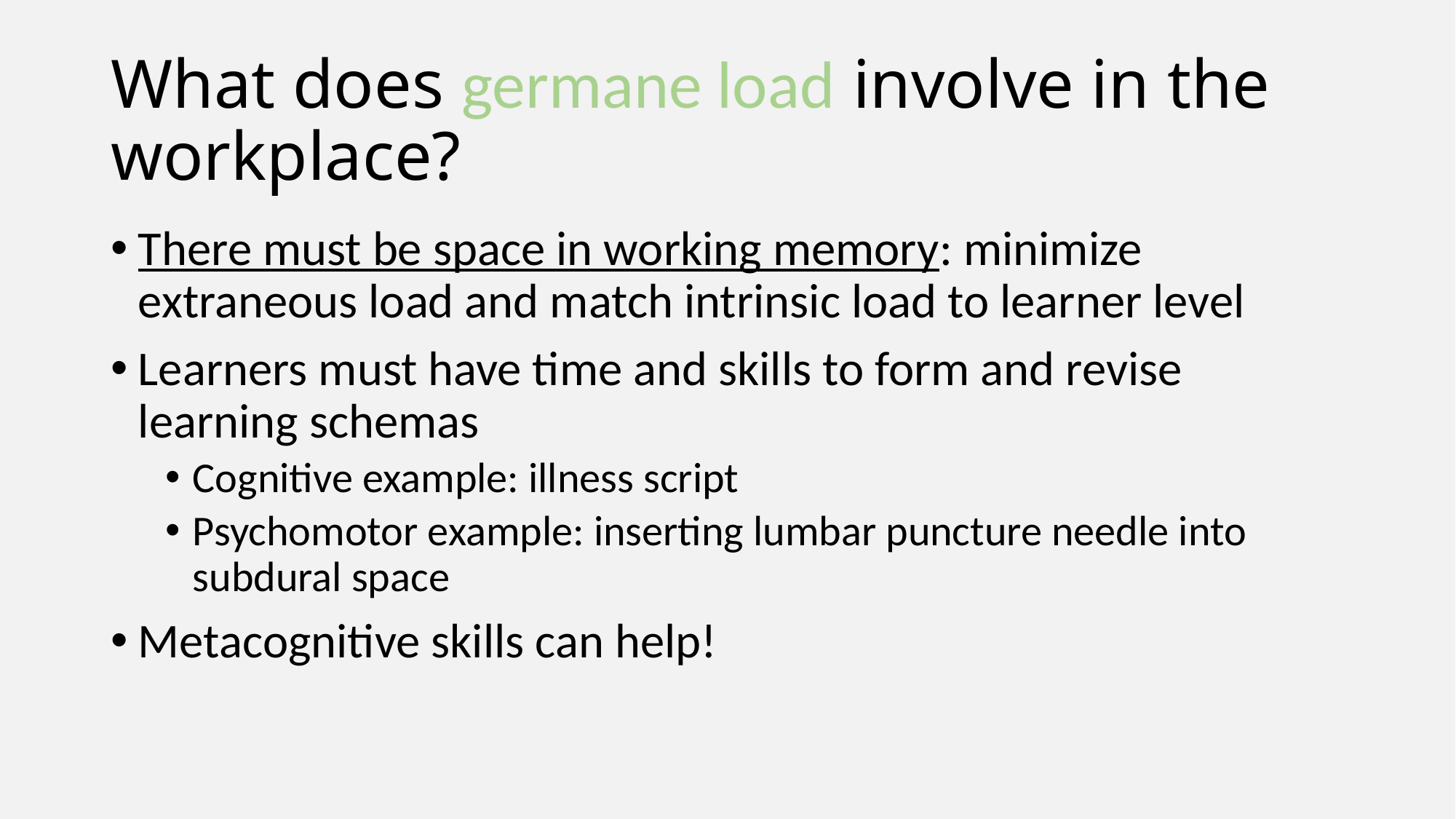

# What does germane load involve in the workplace?
There must be space in working memory: minimize extraneous load and match intrinsic load to learner level
Learners must have time and skills to form and revise learning schemas
Cognitive example: illness script
Psychomotor example: inserting lumbar puncture needle into subdural space
Metacognitive skills can help!

## Slide 23
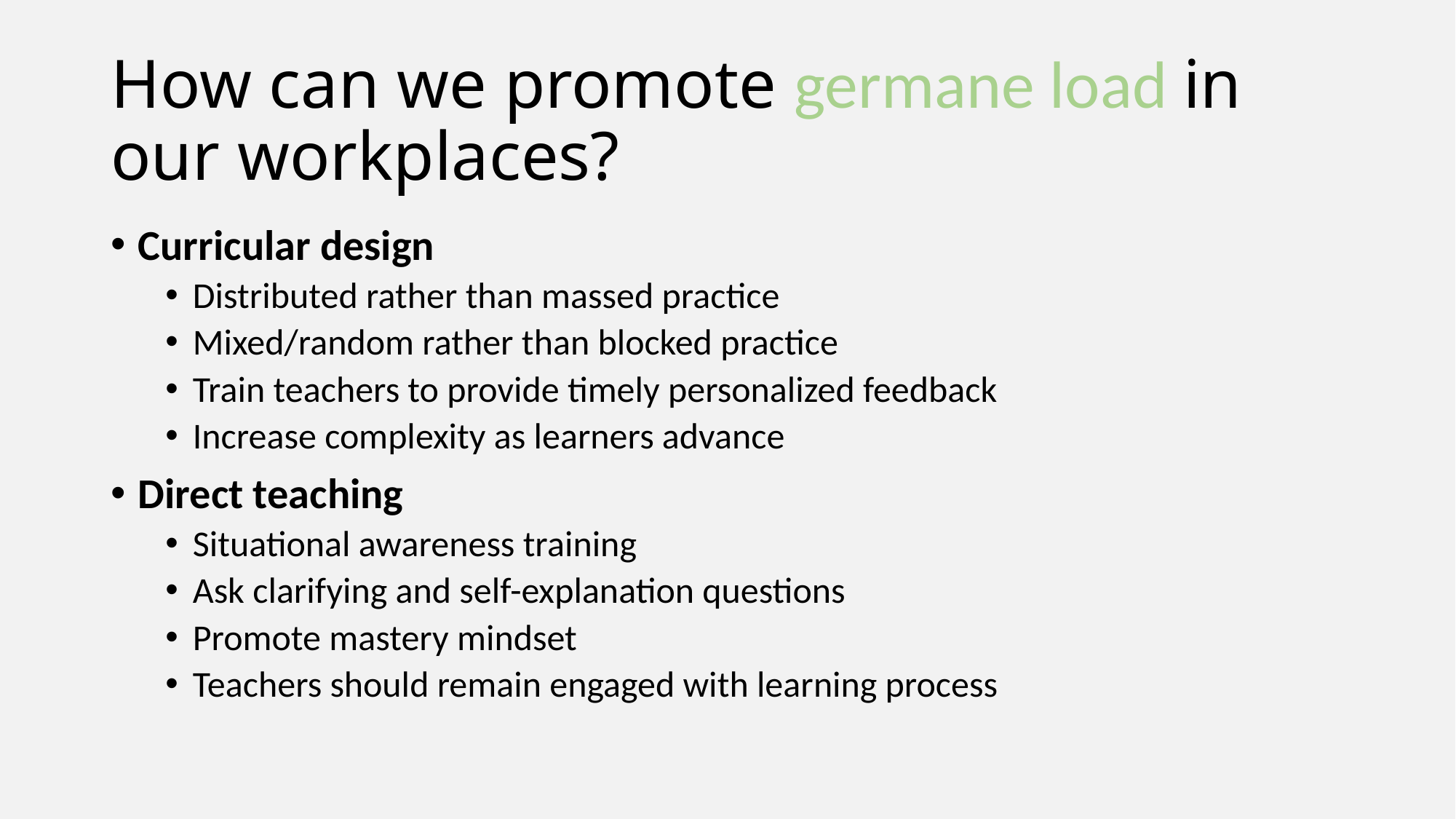

# How can we promote germane load in our workplaces?
Curricular design
Distributed rather than massed practice
Mixed/random rather than blocked practice
Train teachers to provide timely personalized feedback
Increase complexity as learners advance
Direct teaching
Situational awareness training
Ask clarifying and self-explanation questions
Promote mastery mindset
Teachers should remain engaged with learning process

## Slide 24
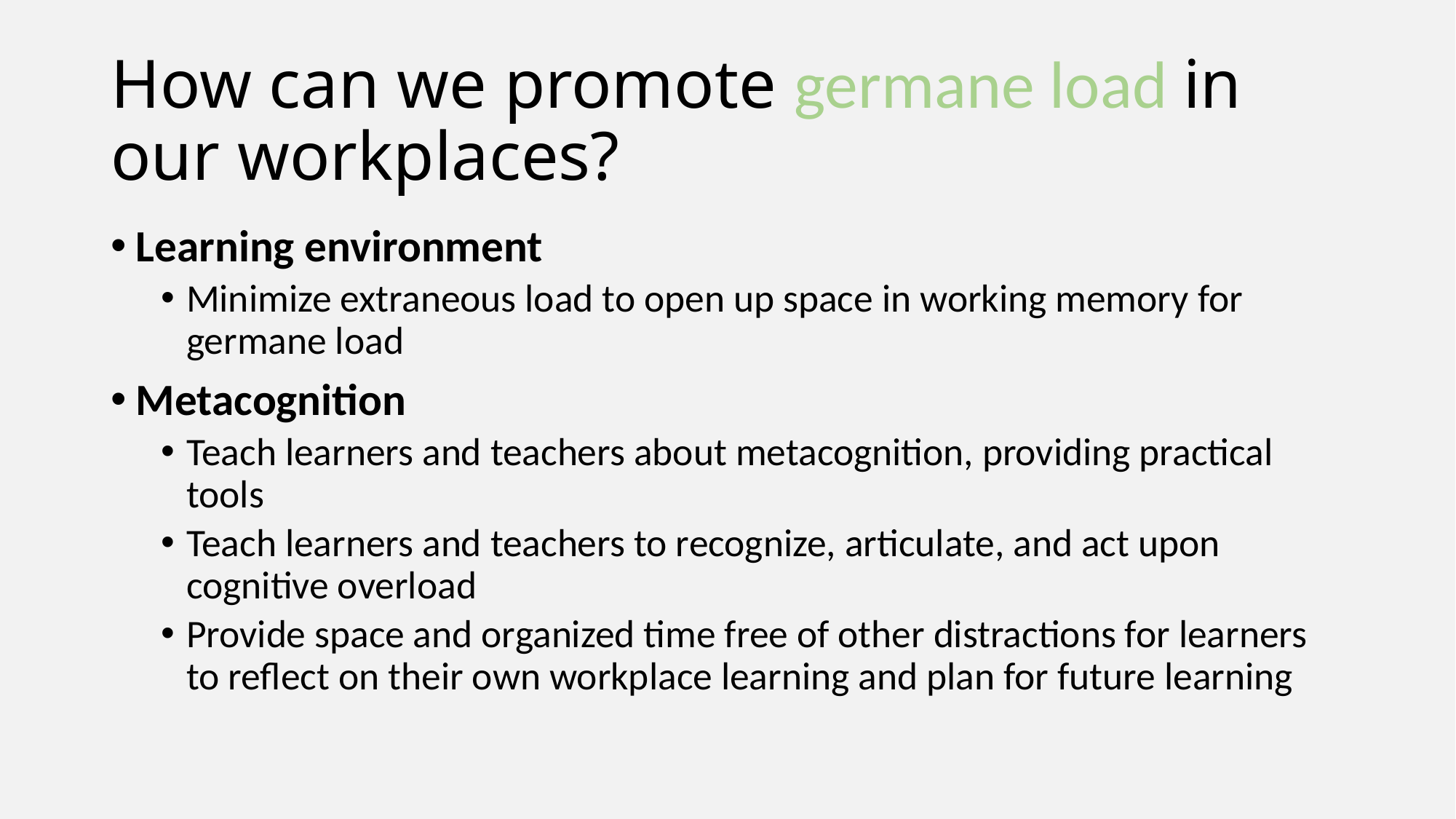

# How can we promote germane load in our workplaces?
Learning environment
Minimize extraneous load to open up space in working memory for germane load
Metacognition
Teach learners and teachers about metacognition, providing practical tools
Teach learners and teachers to recognize, articulate, and act upon cognitive overload
Provide space and organized time free of other distractions for learners to reflect on their own workplace learning and plan for future learning

## Slide 25
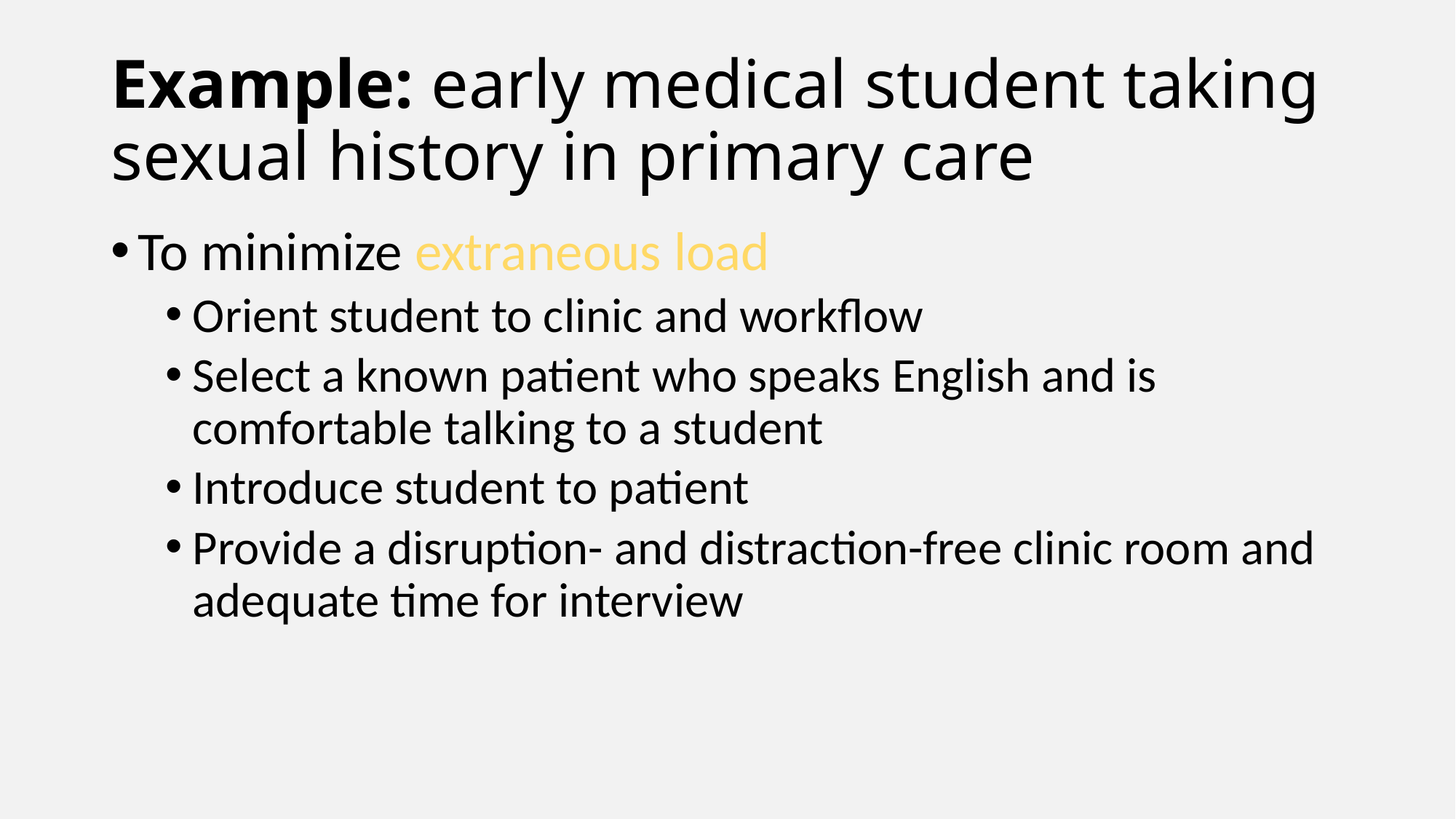

# Example: early medical student taking sexual history in primary care
To minimize extraneous load
Orient student to clinic and workflow
Select a known patient who speaks English and is comfortable talking to a student
Introduce student to patient
Provide a disruption- and distraction-free clinic room and adequate time for interview

## Slide 26
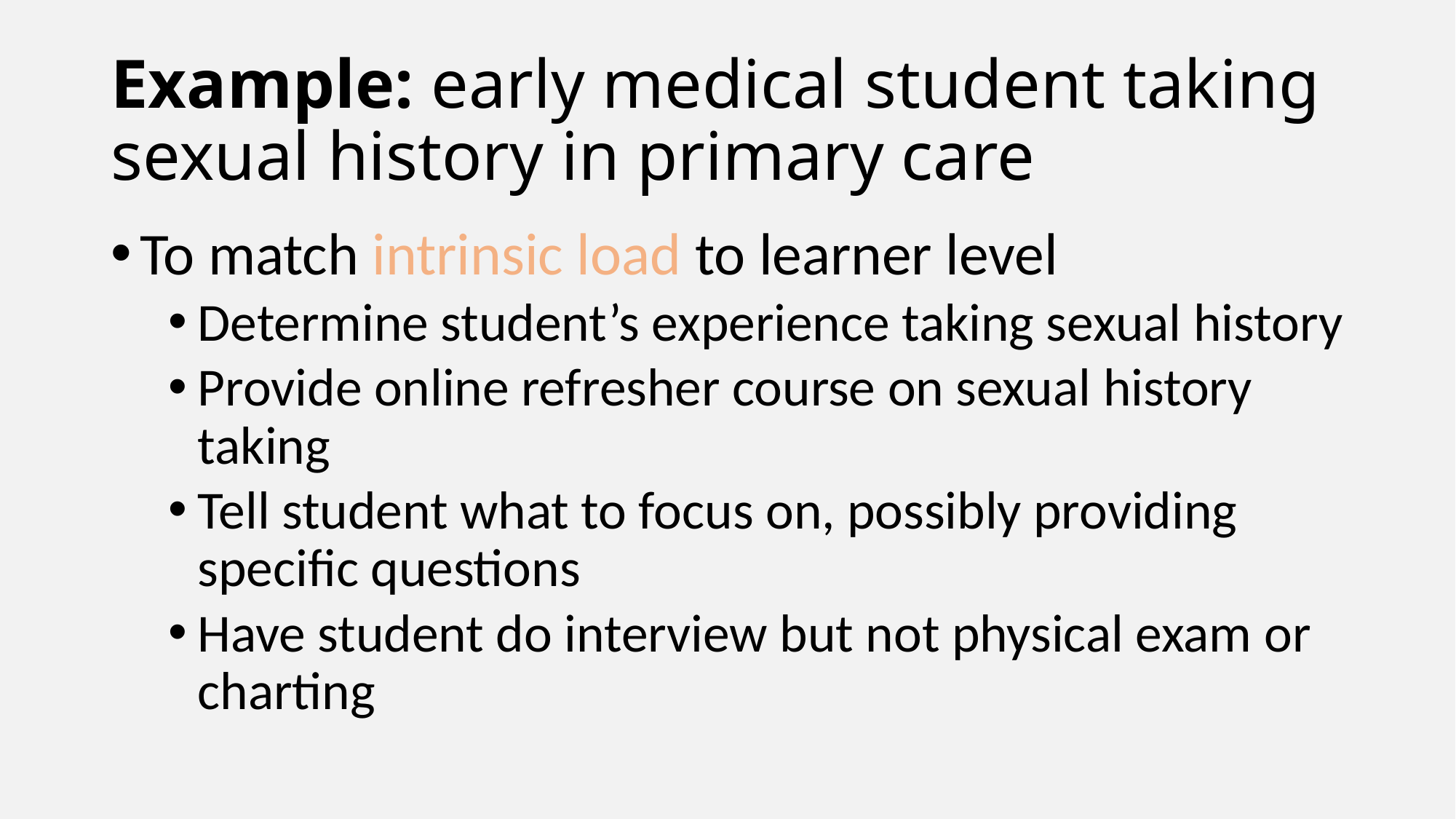

# Example: early medical student taking sexual history in primary care
To match intrinsic load to learner level
Determine student’s experience taking sexual history
Provide online refresher course on sexual history taking
Tell student what to focus on, possibly providing specific questions
Have student do interview but not physical exam or charting

## Slide 27
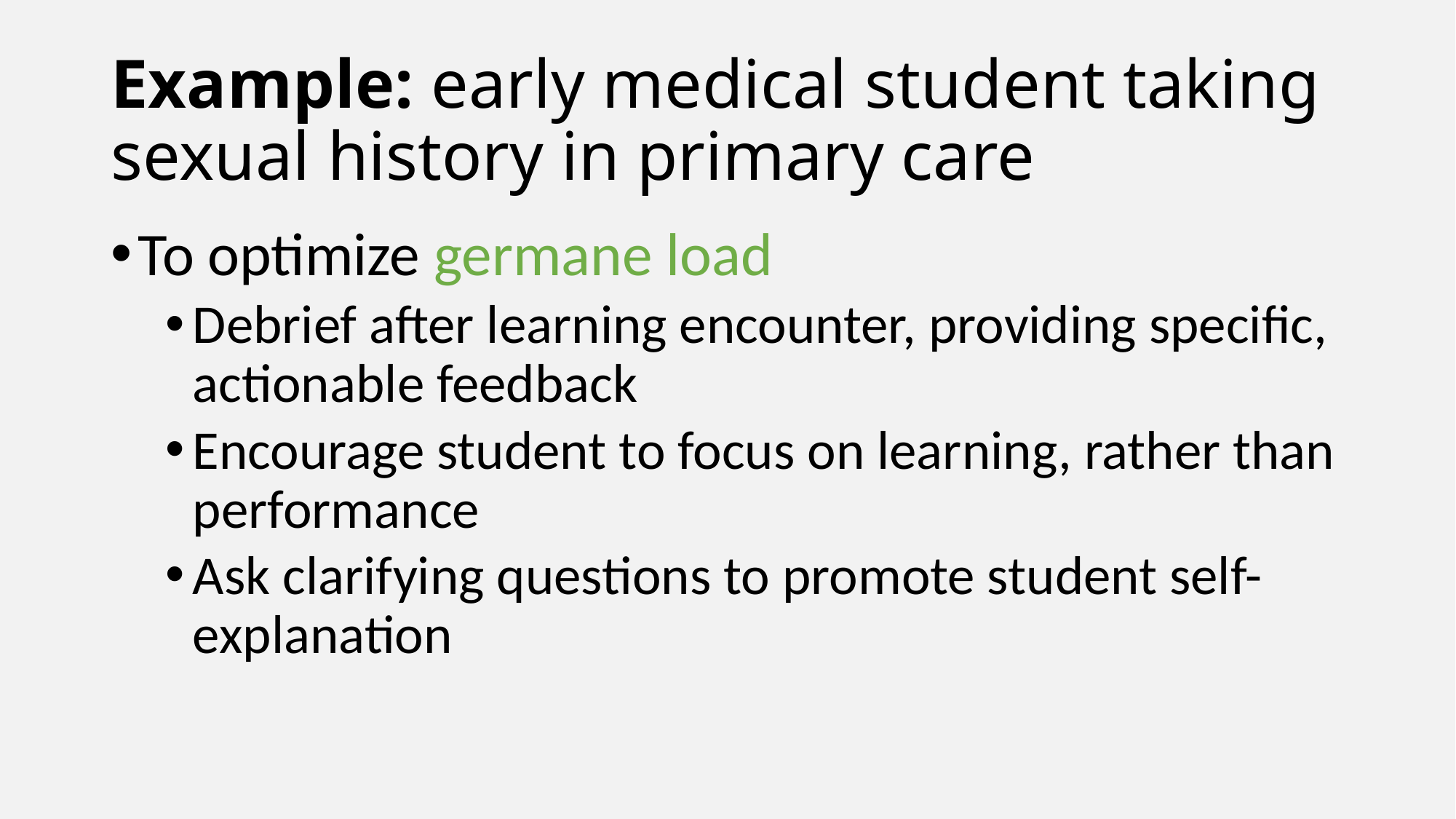

# Example: early medical student taking sexual history in primary care
To optimize germane load
Debrief after learning encounter, providing specific, actionable feedback
Encourage student to focus on learning, rather than performance
Ask clarifying questions to promote student self-explanation

## Slide 28
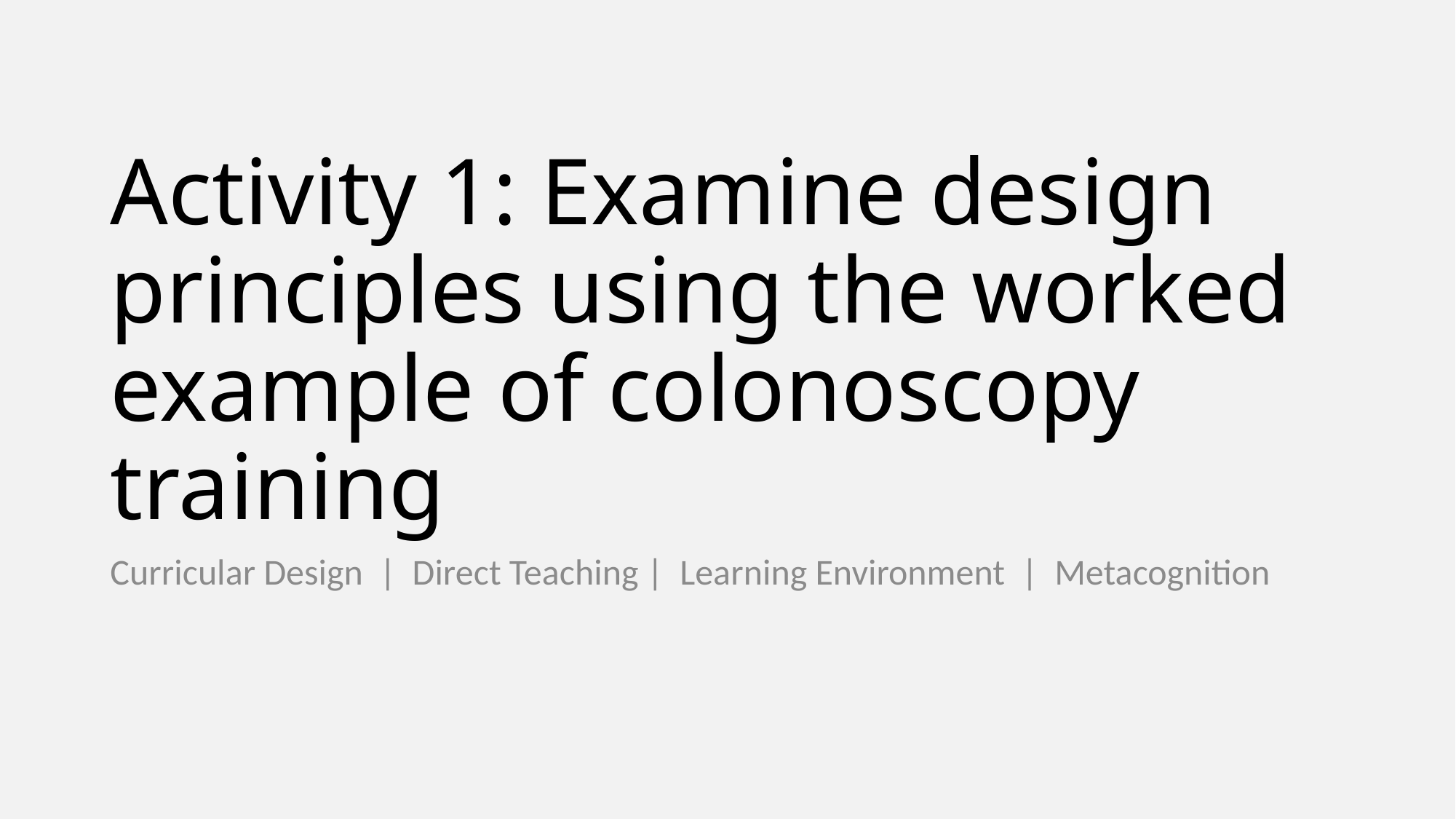

# Activity 1: Examine design principles using the worked example of colonoscopy training
Curricular Design | Direct Teaching | Learning Environment | Metacognition

## Slide 29
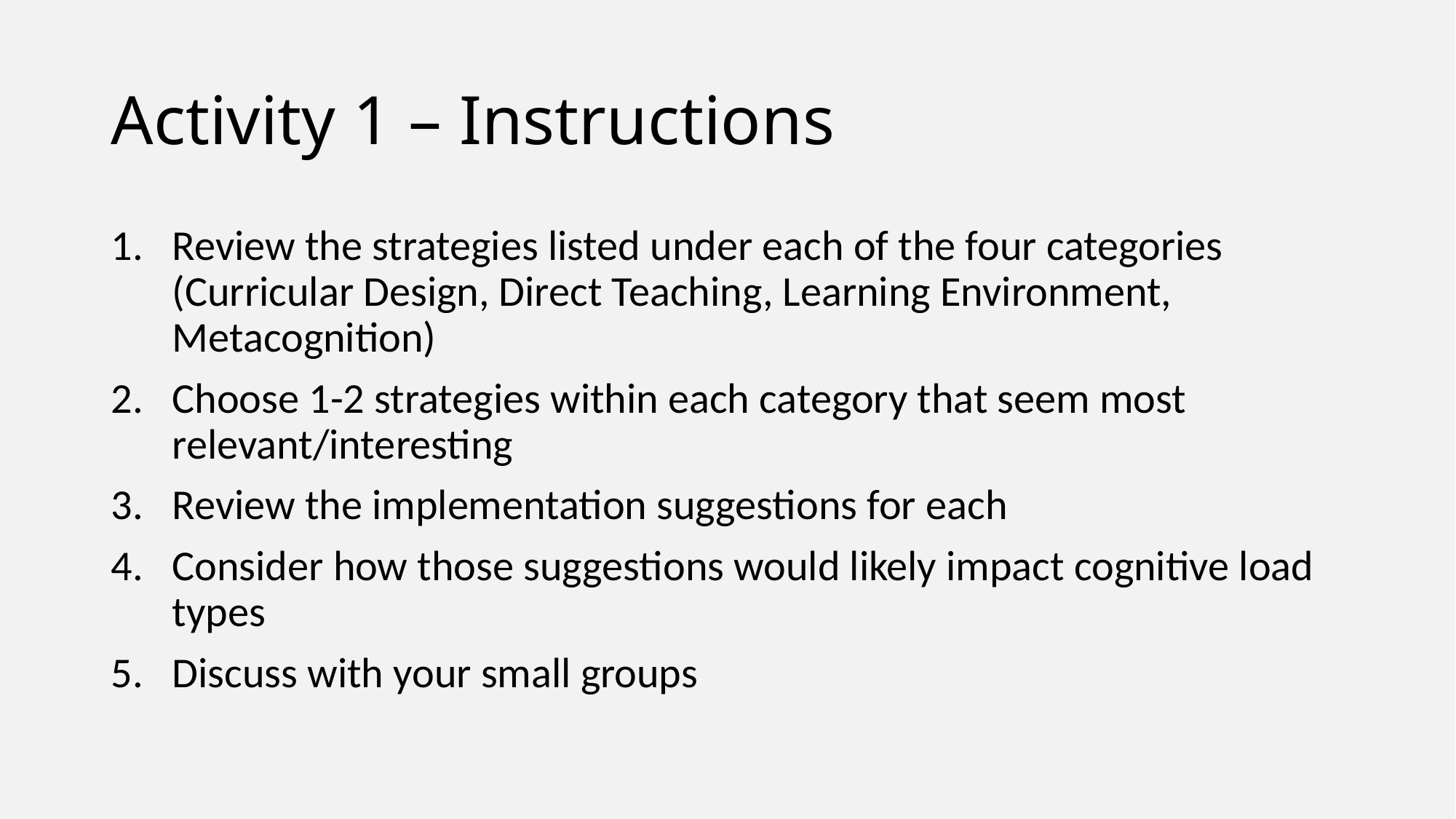

# Activity 1 – Instructions
Review the strategies listed under each of the four categories (Curricular Design, Direct Teaching, Learning Environment, Metacognition)
Choose 1-2 strategies within each category that seem most relevant/interesting
Review the implementation suggestions for each
Consider how those suggestions would likely impact cognitive load types
Discuss with your small groups

## Slide 30
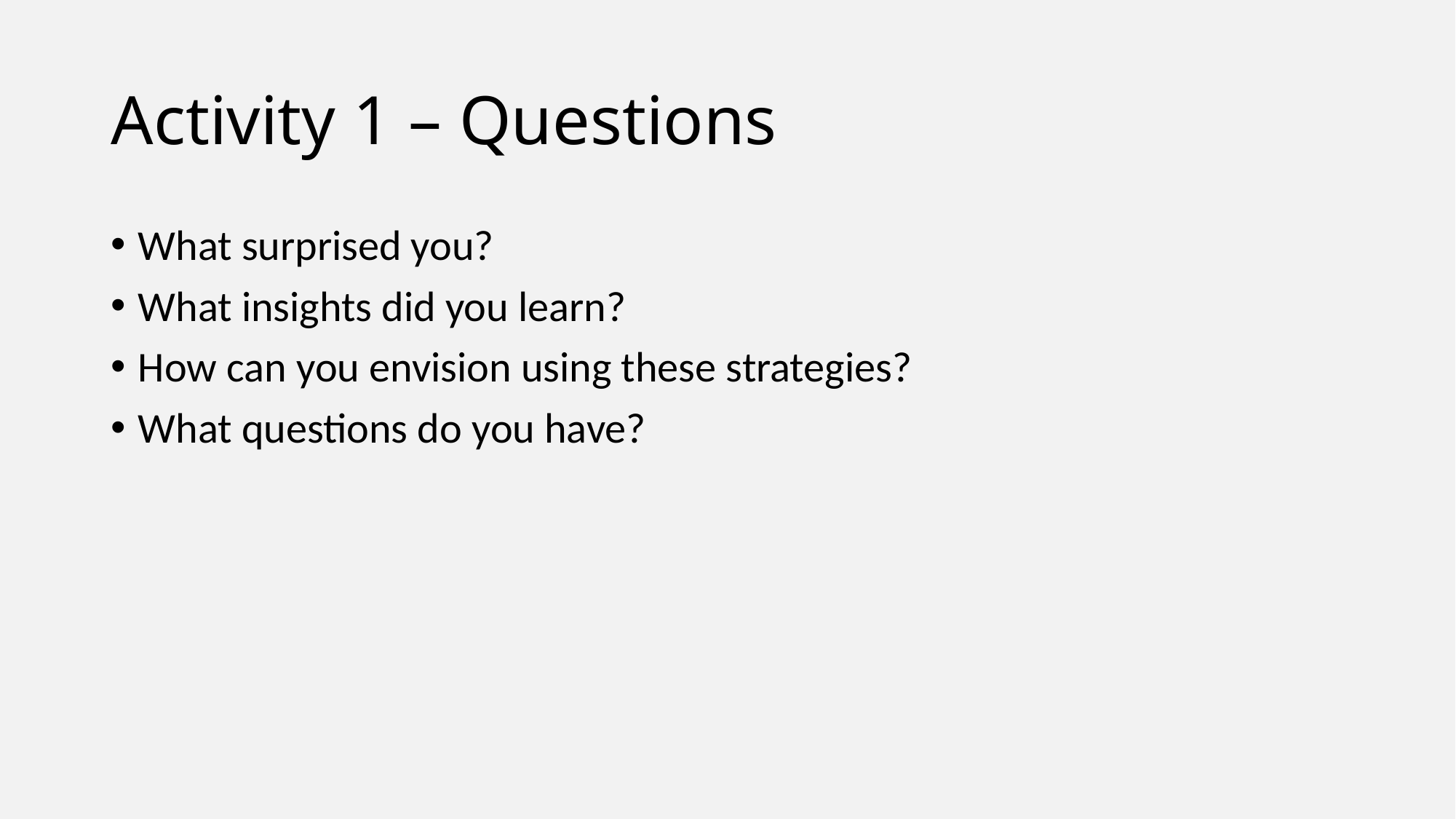

# Activity 1 – Questions
What surprised you?
What insights did you learn?
How can you envision using these strategies?
What questions do you have?

## Slide 31
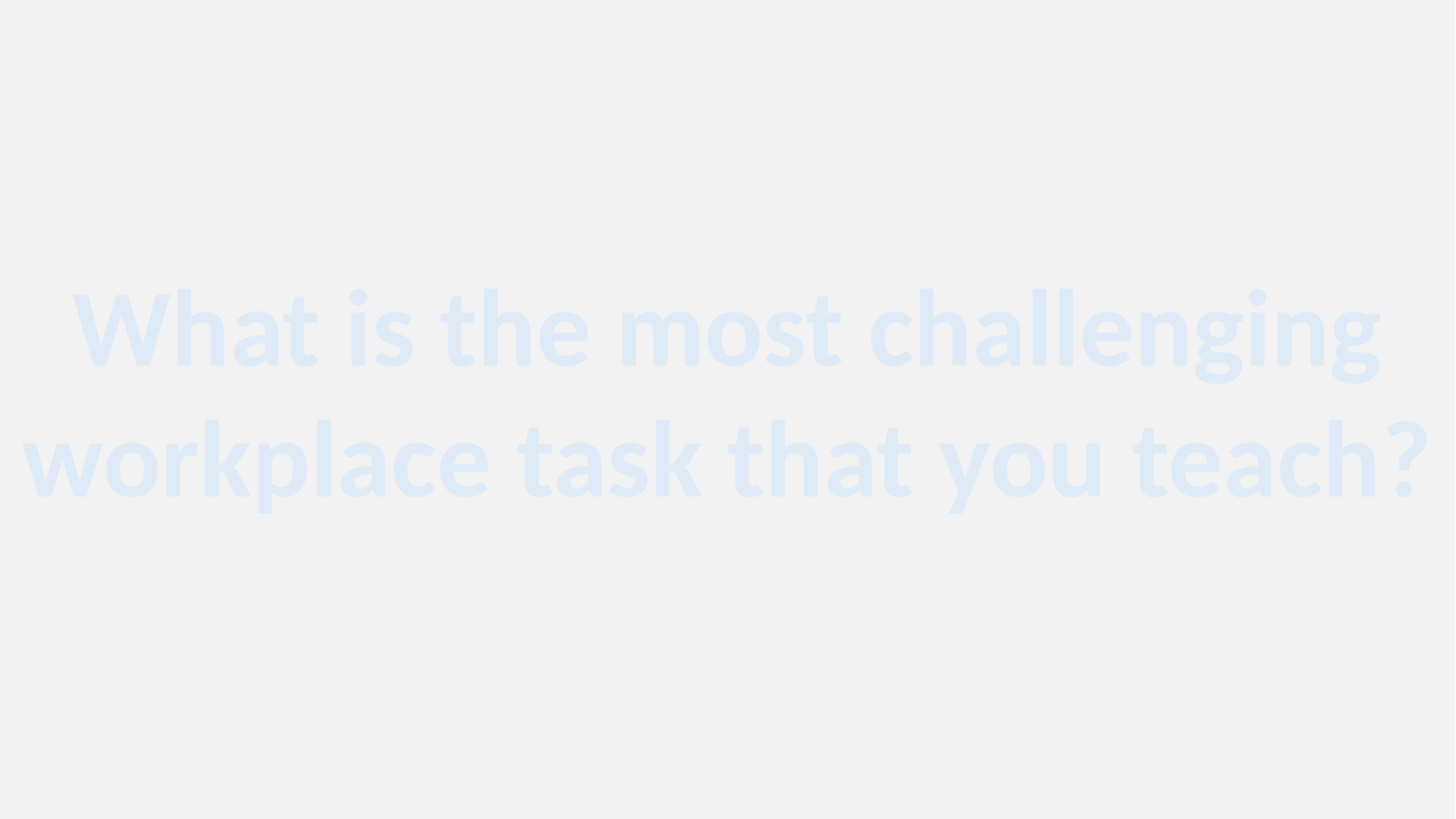

What is the most challenging
workplace task that you teach?

## Slide 32
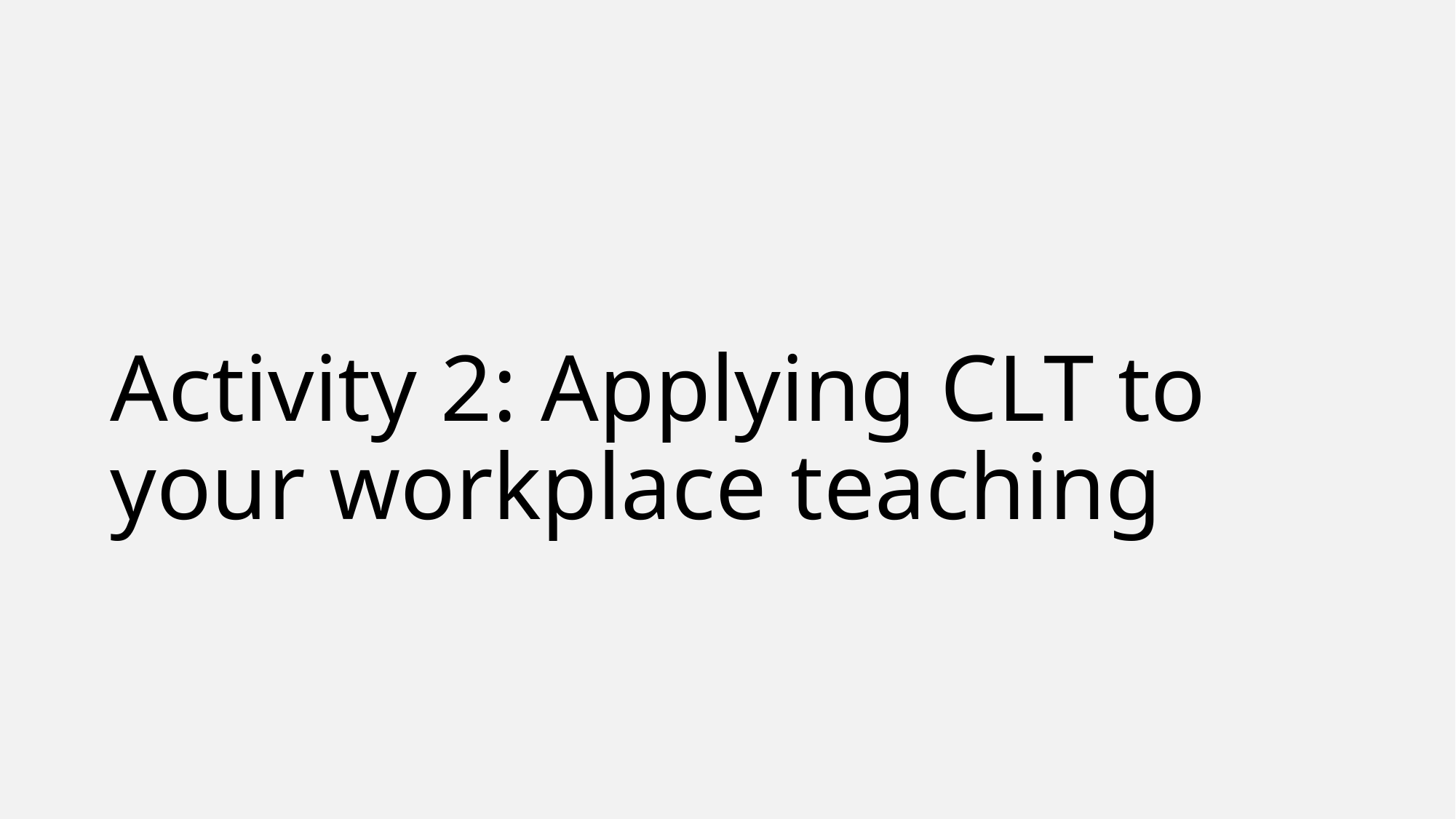

# Activity 2: Applying CLT to your workplace teaching

## Slide 33
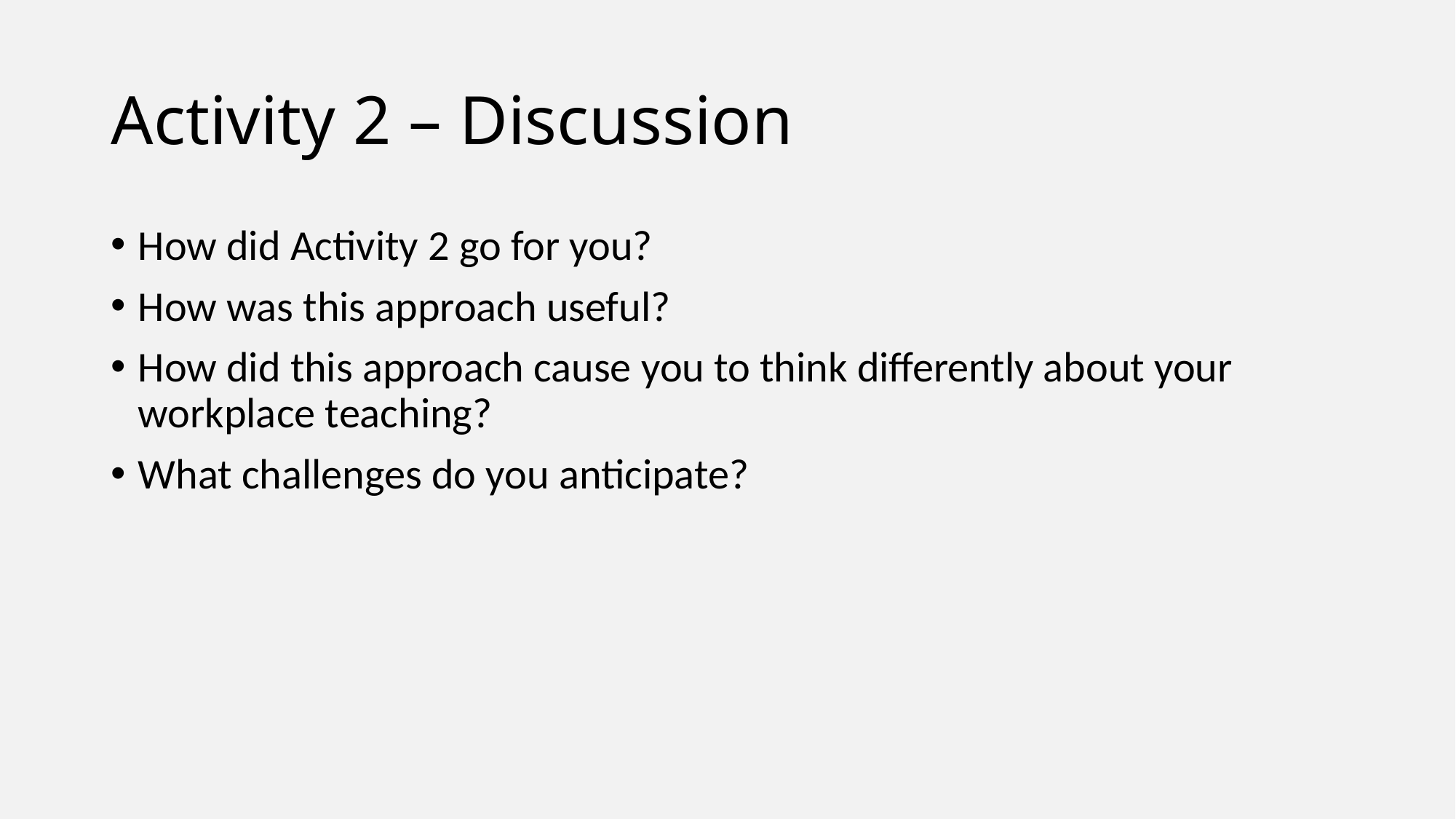

# Activity 2 – Discussion
How did Activity 2 go for you?
How was this approach useful?
How did this approach cause you to think differently about your workplace teaching?
What challenges do you anticipate?

## Slide 34
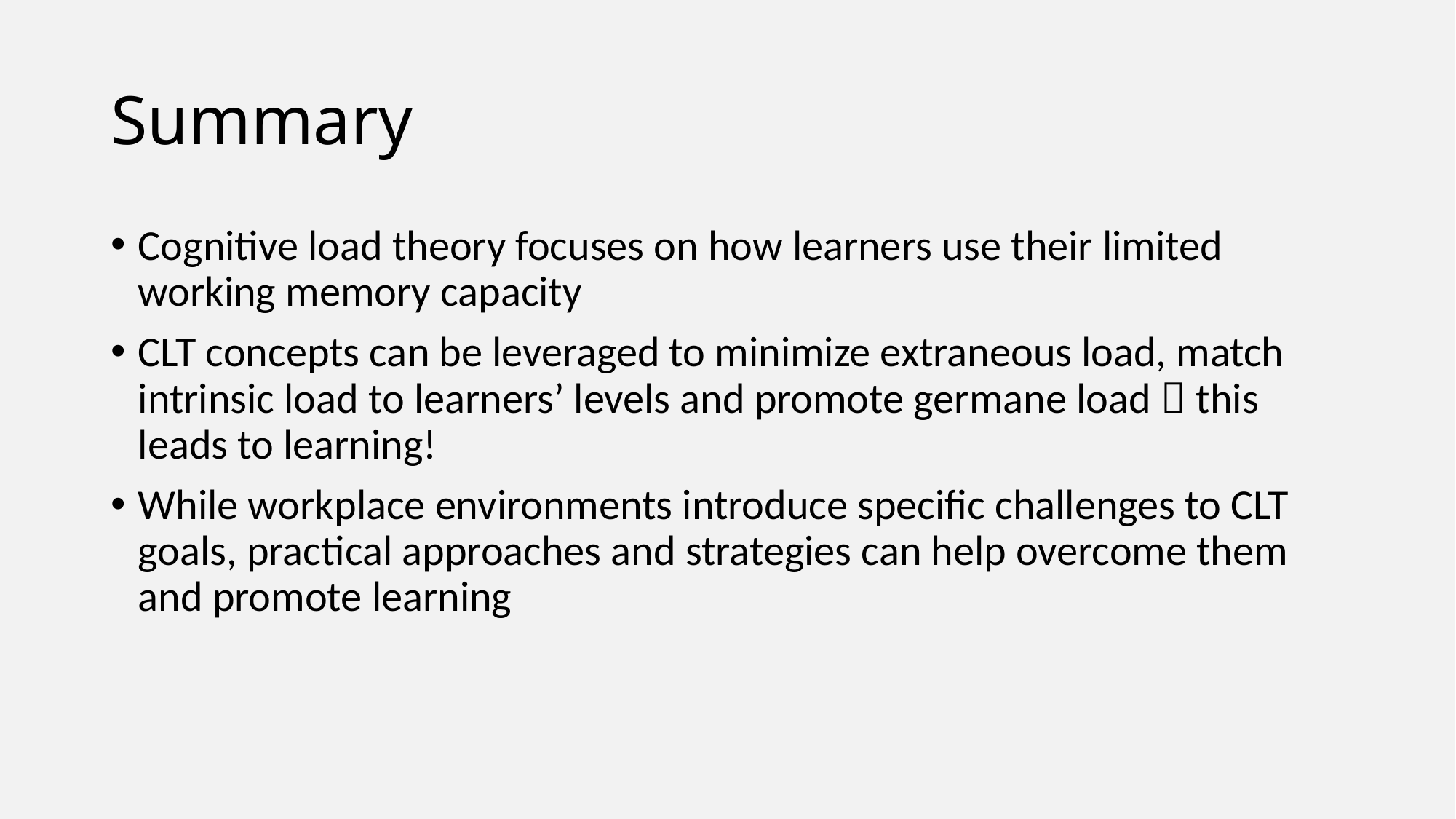

# Summary
Cognitive load theory focuses on how learners use their limited working memory capacity
CLT concepts can be leveraged to minimize extraneous load, match intrinsic load to learners’ levels and promote germane load  this leads to learning!
While workplace environments introduce specific challenges to CLT goals, practical approaches and strategies can help overcome them and promote learning
