# Supplementary material for: Using Cognitive Load Theory to Improve Teaching in the Clinical Workplace
Source: MedEdPORTAL. 2020 Oct 2;16:10983. doi: 10.15766/mep_2374-8265.10983 (PMC7549387; doi:10.15766/mep_2374-8265.10983)
Supplement: Supplementary file 1 — Large-Group CLT Overview.pptxActivity 1 Small-Group Worked Example.docxActivity 2 Individual Activity Design.docxWorkshop Participant Evaluations.docxFollow-Up Survey.docxFacilitator Guide.docx [file mep_2374-8265.10983-s001.zip › B. Activity 1 Small-Group Worked Example.docx]

**Appendix B – Small-Group**

**For Activity 1:**

- With those in your group, review the worked example of how to apply CLT to the exemplar setting of colonoscopy training.
- After reviewing the worked example, discuss the following questions in your group:
  - What surprised you?
  - What insights did you learn?
  - How can you envision using these strategies?
  - What questions do you have?
- After 20 minutes’ time, we will report out and discuss.

| **Strategies** | **What are a few potential ways to implement in your workplace teaching?** | **Could these practices: (Yes/No/Maybe)** | | |
| --- | --- | --- | --- | --- |
|  |  | **Reduce EL?** | **Match IL?** | **Optimize GL?** |
| **Curricular Design** |  |  |  |  |
| Ensure overall CL or intrinsic load of learning setting is neither *too high* nor *too low* | - Develop system to monitor and report on fellows’ competence so teachers are aware of their current level - Have dedicated check-in prior to each endoscopy session to review competence and set expectations for amount of procedure fellow will complete, when attending will take over; and review cases for the day to see if any are particularly complex - Have dedicated advanced procedural skills rotations for more advanced fellows |  | Yes | Maybe |
| Use simulation for early learners, especially for complex tasks and those with risk to patients | - Schedule a colonoscopy bootcamp at the beginning of fellowship and require a particular level of performance before starting to participate in procedures on actual patients - Use simulators or real equipment outside of patients to ensure familiarity with equipment and how to use it | Maybe | Yes | Maybe |
| Appraise workplaces to identify areas/tasks with high potential for cognitive overload | - Review setup and workflow of endoscopy unit - Review forms and documents that learners must use - Review usability of EHR and endoscopy documentation system | Yes | Yes | Maybe |
| Standardize common tasks, providing supports when needed | - Create, disseminate and monitor adherence to common or complex tasks in the endoscopy unit - Enable fellows to take lead in QI processes within the endoscopy unit |  | Yes | Maybe |
| Design curricula to support workplace learning that scaffold tasks, gradually increasing complexity and reducing support | - Start with simulation (as above) - Follow dedicates series of partial tasks and gradually build up to whole tasks when competence achieved on partial tasks |  | Yes | Yes |

|  | - Use didactic and small group learning to teach cognitive aspects of endoscopy - Provide endoscopy teachers with techniques/strategies to provide higher-support to early learners and lower-support to more advanced learners |  |  |  |
| --- | --- | --- | --- | --- |
| Facilitate mixed or random practice over block practice | - Design fellow endoscopy schedule so that they perform a variety of procedures (both basic and advanced) during each endoscopy session, or each week - Avoid long stretches of time during which fellows do not participate in endoscopy - Intersperse actual workplace tasks with periods of simulation, or applications like Qstream that can test cognitive knowledge over a specified period of time |  | Yes | Yes |
| **Direct Teaching** |  |  |  |  |
| Teacher should remain engaged with learning, limiting tangential conversations | - Provide peer observation of endoscopy teachers with feedback - Empower fellows to respectfully remind their endoscopy teachers if conversations in the room are distracting the fellow - Include items assessing teacher engagement during endoscopy training in fellows’ evaluations of endoscopy teachers | Yes |  | Yes |
| Teach teachers to monitor for cognitive overload in learners | - Offer faculty development sessions on CLT - Discuss signs of cognitive overload in endoscopy training (sighing, other vocalizations, performing same maneuver repeatedly without success, doing maneuvers that lack clear purpose or intent) |  | Yes | Maybe |
| Attend to learner emotion, especially in crisis situations | - Offer faculty development sessions discussing impact of emotion on learning - Provide faculty with tools to address negative learner emotions - Ensure debriefing and discussion after adverse endoscopy outcome | Yes |  | Maybe |
| **Learning Environment** |  |  |  |  |
| Leverage graphical displays and technology to reduce extraneous load | - Use high-definition endoscopy equipment and monitor - Critically assess utility of endoscopy documentation software | Yes | Yes | Maybe |
| Monitor learning environments for distractions and contextual factors that contribute to extraneous load | - Observe colonoscopies performed across different days and times and identify potential distractions and contextual factors (e.g., non-English speaking patients) (this could be a good area for fellow QI involvement) - Teach attendings and staff to minimize conversations and other distractions when fellows are scoping (especially junior fellows) | Yes |  | Maybe |
| Engineer workplace environments to minimize distractions and redundancy | - Critically assess physical layout, workflow, and procedures for areas of redundancy and distraction that require fellows’ attention | Yes |  | Maybe |

| Monitor for, and mitigate, learner fatigue | - Monitor duty hours - If fellow came in overnight or is otherwise fatigued, reduce endoscopy responsibilities (e.g., attending does intake and/or report, fellow only does part of task, fellow mostly observes rather than performs procedures) | Yes | Maybe | Maybe |
| --- | --- | --- | --- | --- |
| **Metacognition** |  |  |  |  |
| Help learners know where to direct attention/ working memory | - Thoroughly orient endoscopy learners to the tasks required for endoscopic procedures, and the endoscopy unit and its workflow, emphasizing the aspects that are most critical for high- quality, safe endoscopy procedures - Provide faculty development to enable attendings to guide fellows’ attention and use of working memory resources | Yes |  | Yes |
| Teach learners to manage distractions | - Provide a learning session where a consultant discusses techniques to enable focus on task at hand and how to avoid or mitigate negative impact of distractions - Endoscopy teachers should redirect fellows to task at hand when they appear distracted | Yes |  | Maybe |
| Teach learners to monitor their level of cognitive load and communicate feelings of overload | - Teach endoscopy learners about CLT and signs/feelings of cognitive overload - Provide learners with language they can use to indicate feelings of cognitive overload | Maybe | Maybe | Maybe |
| Teach learners to use meta-cognitive techniques to enhance learning | - Offer a learning session on aspects of metacognition with future small group application - Provide endoscopy teachers with prompts to ask during endoscopy to prompt metacognitive activities - Test fellows’ metacognitive skills |  |  | Yes |
