# Supplementary material for: Using Cognitive Load Theory to Improve Teaching in the Clinical Workplace
Source: MedEdPORTAL. 2020 Oct 2;16:10983. doi: 10.15766/mep_2374-8265.10983 (PMC7549387; doi:10.15766/mep_2374-8265.10983)
Supplement: Supplementary file 1 — Large-Group CLT Overview.pptxActivity 1 Small-Group Worked Example.docxActivity 2 Individual Activity Design.docxWorkshop Participant Evaluations.docxFollow-Up Survey.docxFacilitator Guide.docx [file mep_2374-8265.10983-s001.zip › C. Activity 2 Individual Activity Design.docx]

# Appendix C. End-of-Workshop Individual Activity

**Activity 2**

1. **Select and indicate a personally relevant teaching workplace activity you would like to consider, or choose from the following options: patient handoff, central venous catheter insertion, or introduction of new tool in the surgical suite.**
2. **Choose one of the following *Curricular Design* strategies below to apply to the activity that you identified. Describe an approach to implementing this in your workplace setting.**
   - Ensure overall cognitive load or intrinsic load of learning setting is neither too high nor too low
   - Use simulation for early learners, especially for complex tasks and those with risk to patients
   - Appraise workplaces to identify areas/tasks with high potential for cognitive overload
   - Standardize common tasks, providing supports when needed
   - Design curricula to support workplace learning that scaffold tasks, gradually increasing complexity and reducing support
   - Facilitate mixed or random practice over block practice

# Choose one of the following *Direct Teaching* strategies below that apply to the activity that you identified. Describe an approach to implementing this in your workplace setting.

- - Teacher should remain engaged with learning, limiting tangential conversations
  - Teach teachers to monitor for cognitive overload in learners
  - Attend to learner emotion, especially in crisis situations

# Choose one of the following *Learning Environment* strategies below that apply to the activity that you identified. Describe an approach to implementing this in your workplace setting.

- - Leverage graphical displays and technology to reduce extraneous load
  - Monitor learning environments for distractions and contextual factors that contribute to extraneous load
  - Engineer workplace environments to minimize distractions and redundancy
  - Monitor for, and mitigate, learner fatigue

# Choose one of the following *Metacognition* strategies below that apply to the activity that you identified. Describe an approach to implementing this in your workplace setting.

- - Help learners know where to direct attention/ working memory
  - Teach learners to manage distractions
  - Teach learners to monitor their level of cognitive load and communicate feelings of overload
  - Teach learners to use meta-cognitive techniques to enhance learning
