# Supplementary material for: Using Cognitive Load Theory to Improve Teaching in the Clinical Workplace
Source: MedEdPORTAL. 2020 Oct 2;16:10983. doi: 10.15766/mep_2374-8265.10983 (PMC7549387; doi:10.15766/mep_2374-8265.10983)
Supplement: Supplementary file 1 — Large-Group CLT Overview.pptxActivity 1 Small-Group Worked Example.docxActivity 2 Individual Activity Design.docxWorkshop Participant Evaluations.docxFollow-Up Survey.docxFacilitator Guide.docx [file mep_2374-8265.10983-s001.zip › D. Workshop Participant Evaluations.docx]

**Workshop Participant Evaluation**

*University of San Francisco Workshop, April 30, 2018*

Please fill in the circle **completely**

for the rating you choose

Poor Fair Good

Very

Excellent

| ● |  |  |  | Good |  |
| --- | --- | --- | --- | --- | --- |
| 1. Advance communication | O | O | O | O | O |
| 2. Clarity of objectives | O | O | O | O | O |
| 3. Session organization | O | O | O | O | O |
| 4. Usefulness of materials (slides, handouts, etc.) | O | O | O | O | O |
| 5a. Overall quality of teaching by the facilitator A | O | O | O | O | O |
| 5b. Overall quality of teaching by facilitator B | O | O | O | O | O |
| 6. Overall quality of session | O | O | O | O | O |
| 7. Please estimate the likelihood that you will make changes in your teaching/professional practices as a result of this CME activity. | O | O | O | O | O |

1. Please list the changes you will make to your teaching/professional practice.
2. What aspects of the session did you find most **valuable**?

**Continued next page . . .**

1. How could the session be **improved**? Please include comments on convenience of session time and location.
2. In which educational areas will you plan to change your educational practice as a result of this activity? Please check all that apply:

▢ Application of learning theories (1)

▢ Assessment (2)

▢ Career and leadership (3)

▢ Curriculum development (4)

▢ Education research (5)

▢ Teaching methods (6)

▢ Other (please describe): (7)

1. Please identify any barriers you perceive in implementing these changes (check all that apply):

▢ Current educational skills and knowledge (2)

▢ Lack of support from leadership and/or colleagues (3)

▢ Lack of resources (4)

▢ Time (1)

▢ None / Not applicable (6)

Other (please describe): (5)

1. What **topics** do you suggest for future faculty development workshops and grand rounds?

Please fill in the circle **completely**

for the rating you choose ●

1. Do you agree with the following statement: “This course was free of commercial bias”?

YES NO

O O

If “NO,” explain why.

**Workshop Participant Evaluation**

*University of Texas Workshop on May 11, 2018*

| **PLEASE RATE THE PLENARY SPEAKER:** | Strongly Disagree | Disagree | Neutral | Agree | Strongly Agree |
| --- | --- | --- | --- | --- | --- |
| The presenter communicated clearly & effectively. | 🞏 | 🞏 | 🞏 | 🞏 | 🞏 |
| Information presented was relevant to me. | 🞏 | 🞏 | 🞏 | 🞏 | 🞏 |
| Audiovisuals and/or handouts were used effectively. | 🞏 | 🞏 | 🞏 | 🞏 | 🞏 |
| What did you like about the Plenary Talk or overall symposium?  What recommendations do you have? | | | | | |

**Workshop/Oral/Small Group Discussion Evaluation Form**

**NAME OF FACILITATOR / PRESENTER**

| **PLEASE RATE THE QUALITY OF THE SESSION YOU ATTENDED:** | Strongly Disagree | Disagree | Neutral | Agree | Strongly Agree |
| --- | --- | --- | --- | --- | --- |
| I learned valuable information/tools/ideas that can be applied to my work**.** | 🞏 | 🞏 | 🞏 | 🞏 | 🞏 |
| The educational content of this session was useful. | 🞏 | 🞏 | 🞏 | 🞏 | 🞏 |
| Presenter(s) communicated clearly & effectively. | 🞏 | 🞏 | 🞏 | 🞏 | 🞏 |

WHAT DID YOU LIKE ABOUT THIS SESSION?

WHAT RECOMMENDATIONS DO YOU HAVE FOR THE PRESENTER?
