# Supplementary material for: Using Cognitive Load Theory to Improve Teaching in the Clinical Workplace
Source: MedEdPORTAL. 2020 Oct 2;16:10983. doi: 10.15766/mep_2374-8265.10983 (PMC7549387; doi:10.15766/mep_2374-8265.10983)
Supplement: Supplementary file 1 — Large-Group CLT Overview.pptxActivity 1 Small-Group Worked Example.docxActivity 2 Individual Activity Design.docxWorkshop Participant Evaluations.docxFollow-Up Survey.docxFacilitator Guide.docx [file mep_2374-8265.10983-s001.zip › E. Follow-Up Survey.docx]

# Appendix E. Follow-up survey.

**First, we would like to ask you several questions about the different types of cognitive load. Please do not use any external resources when answering these questions. Instead, respond based on what you remember from the workshop. *(Note: for publication purposes, correct answer indicated with an asterisk below.)***

1. A pharmacy student diligently listens and activates prior knowledge to link new information with her existing knowledge while working in the hospital's transplant pharmacy. *What type of cognitive load is occurring in this pharmacy student?*
   1. Extraneous load
   2. Germane load*
   3. Intrinsic load
2. According to cognitive load theory, this type of cognitive load should be:
   1. Matched to the competence of the learner
   2. Minimized
   3. Optimized*
3. A nursing student has difficulty learning on ward rounds because of texts and Twitter updates he receives on his phone, and tangential conversations he overhears among the hospital staff. *What type of cognitive load is this nursing student experiencing?*
   1. Extraneous load*
   2. Germane load
   3. Intrinsic load
4. According to cognitive load theory, this type of cognitive should load be:
   1. Matched to the competence of the learner
   2. Minimized*
   3. Optimized
5. A medical intern rotating in the ICU feels overwhelmed by the amount and complexity of information she has to manage. *What type of cognitive load is causing this medical student to feel overwhelmed?*
   1. Extraneous load
   2. Germane load
   3. Intrinsic load*
6. According to cognitive load theory, this type of cognitive should load be:
   1. Matched to the competence of the learner*
   2. Minimized
   3. Optimized

# Next, we would like to hear how you thought about and/or utilized the information that we discussed during the workshop you attended.

1. How (if at all) did the information about cognitive load presented during the workshop impact how you think about training health professions learners (e.g., students, residents, fellows) in workplace settings? *[Text box for response]*
2. Have you planned or made any changes to your workplace teaching or curriculum as a result of learning about cognitive load theory during the workshop? *[Yes/No]*
3. *[If response to 8 is Yes]:* What changes have you planned or made? Please provide as much detail as you are willing, and be sure to indicate the setting (e.g., ambulatory clinic, inpatient ward, operating room, simulation), and the type and level of learners involved (e.g., pharmacy student, surgical resident, gastroenterology fellow).
4. *[If response to 8 is Yes]:* Which of the following cognitive load goals do you think this change could impact (select all that apply)?
   1. Minimize extraneous load
   2. Match intrinsic load to the learner’s competence
   3. Optimize germane load
5. *[If response to 8 is Yes]:* Did you experience any barriers or challenges in making these teaching or curricular changes? If so, please describe. *[Text box for response]*
6. *[If response to 8 is No]:* Why have you not planned or made any changes to your workplace teaching after learning about cognitive load theory during the workshop? For example, did you feel cognitive load was irrelevant to your teaching setting? Did you feel you lacked skills or support to implement teaching changes? Did you not have time to make any changes? Please be candid in your response.
7. Please rate your familiarity with cognitive load theory at this time. *[0-100 slider with left anchor of ‘very unfamiliar’ and right anchor of ‘very familiar’].*
