# Supplementary material for: Using Cognitive Load Theory to Improve Teaching in the Clinical Workplace
Source: MedEdPORTAL. 2020 Oct 2;16:10983. doi: 10.15766/mep_2374-8265.10983 (PMC7549387; doi:10.15766/mep_2374-8265.10983)
Supplement: Supplementary file 1 — Large-Group CLT Overview.pptxActivity 1 Small-Group Worked Example.docxActivity 2 Individual Activity Design.docxWorkshop Participant Evaluations.docxFollow-Up Survey.docxFacilitator Guide.docx [file mep_2374-8265.10983-s001.zip › F. Facilitator Guide.docx]

# Appendix F – Facilitator guide.

**Personnel:**

- At least two facilitators are recommended for up to 20 participants. If more than 20 participants are present, recommend 1 facilitator per 10 participants.
- Facilitators must be familiar with cognitive load theory (CLT).
- Aligning profession and discipline of participants and facilitators could benefit participants due to awareness of a particular context and ability to provide relevant examples.
- Facilitators should be facile with use of standard computer hardware and software, and, if using web-based tools such as Qualtrics®.

# Equipment:

- Room of adequate size to permit participants to break out into groups of 3-4 with adequate space between groups to facilitate discussion.
- Computer with Microsoft Office suite and projector.
- Participants should bring mobile device with internet access. Tablet or laptop computer preferred as smart phone screens are so small as to make Activity 1 difficult.
- Consider bringing a paper copies of Activity 1 for participants who need them.
- Activity 2 will be enhanced through use of electronic survey software such as Qualtrics®, but is feasible via Word or paper if needed.

# Resources:

1. Large group CLT overview PowerPoint slides
2. Activity 1 (individual worked example activity)
3. Activity 2 (individual personal application activity)
4. To promote facilitator understanding of CLT, see:
   1. Young JQ et al. Cognitive Load Theory: implications for medical education: AMEE Guide No. 86^1^
   2. Sewell JL et al. Cognitive load theory for training health professionals in the workplace: A BEME review of studies among diverse professions: BEME Guide No. 53^2^

# Learning objectives:

By the end of the workshop, learners will be able to:

1. Describe the Atkinson-Shiffrin model of human memory and basic tenets of cognitive load theory.
2. Describe the three types of cognitive load: intrinsic, germane and extraneous cognitive load and give workplace examples of each.
3. Suggest ways to match intrinsic load to learners’ levels of experience in their own workplace setting.
4. Suggest ways to promote optimal germane load among learners within their own workplace teaching setting.
5. Suggest ways to diminish or mitigate potential sources extraneous load in their own workplace setting.

# Conceptual framework:

Cognitive load theory (CLT) is a cognitive learning theory relevant to a wide variety of educational settings in the health professions. The theory focuses on the limited capacity of working memory compared with the significantly greater bandwidth of sensory input and long-term memory.1 The theory outlines how factors intrinsic or extraneous to a given learning task vie for that bandwidth. CLT delineates three specific types of cognitive load: intrinsic load, germane load and extraneous load.3 Intrinsic load occurs as learners perform the essential steps of the learning task itself. Germane load occurs as learners create and modify cognitive schemas that are stored in long term memory for later retrieval and use. Extraneous load occurs when learners use working memory to focus on anything unrelated to task completion or learning; examples include task design (e.g., lack of integration between visual and verbal information) and environment factors, both internal (e.g., negative emotions) and external (e.g., noise). Learning is optimized when intrinsic load is matched to learners’ experience level and extraneous load is minimized; in the motivated learner this will promote working memory space for activities contributing to germane load and therefore learning.1

Just as other learning theories have demonstrated positive impact on clinical teaching,^4^ CLT principles imply practical strategies to optimize learning within health professions education (HPE) settings.^2,5^ In clinical workplace teaching, extraneous load may be minimized by: designing learning environments that minimize distractions, disruptions and multitasking; optimizing usability of visual resources like informational displays and computer interfaces; and properly orienting trainees to learning settings and tasks.6 Additionally, self-regulatory or metacognitive approaches may help learners manage stress and negative emotions to reduce their contributions to extraneous load.7 Intrinsic load can be optimized by designing curricula that adapt learning task difficulty to a learner’s skill level and by providing tools for teachers to familiarize themselves with learners’ prior experience and competence. Simulation can be used to simplify tasks or break tasks into component parts (i.e., part-task approach), which is particularly useful when teaching novice learners.5 A scaffolded curriculum allows learners to repeat tasks with decreasing amounts of support as they gain competence; a practical example of this is the 4C/ID model.8 Germane load can be promoted through increased teacher engagement, interactive questioning of learners, encouraging reflection, and improving learner concentration and metcognition.^9^ In a systematic review of cognitive load in professional workplace settings, the few studies that tested interventions to optimize cognitive load tended to demonstrate benefit.2

**Session time:** The workshop was designed for a 2-hour timeframe, which we consider the minimum adequate time. If a longer timeframe is available, we recommend lengthening each portion accordingly.

# Session components and implementation:

- **Introduction (30 minutes):** Facilitators provide an overview of CLT using the PowerPoint slides with speaker notes (resource A).
- **Activity 1 (20 minutes):** Participants use a worked example to examine CLT design principles. Participants should break out into groups of 3-4 and work together to complete the Activity 1 worksheet (resource B). During this time, facilitators should circulate around the room to answer any questions, and when appropriate, to engage in the small group discussions. Activity 1 instructions for participants are in the slide deck (resource A). The speaker notes provide guidance as to how to walk through the worked example.
- **Activity 1 discussion (10 minutes):** Facilitators lead a discussion of what participants learned from Activity 1. Example questions to promote discussion are in the slide deck (resource A).
- **Activity 2 (20 minutes):** Participants select a personally relevant workplace teaching activity and consider how they can leverage CLT concepts to improve this teaching within four domains: Curricular Design, Direct Teaching, Learning Environment and Metacognition. During this time, facilitators should circulate around the room to answer any questions, and when appropriate, to engage in discussion. This portion works best if a web-based survey platform such as Qualtrics® is used – in this case, Qualtrics® can be programmed to send participants’ responses to them via email. However, pen-and-paper or PDF/MS Word would also work.

**Suggestions for improvement:** Based on our experience implementing this workshop and reviewing evaluations, we recommend considering the following modifications to promote better learning.

- If feasible, ask participants to review basic information about cognitive load theory before attending the workshop. This would permit deeper application of the information using a flipped classroom model. Excellent resources are available in the form of an AMEE Guide^1^ and a BEME review^2^. However, based on personal experience we recognize that participants may not have time to prepare.
- Provide ample time for dialogue and answering questions, which may require increasing the length of the workshop. Consider soliciting questions anonymously (either written on slips of paper or using software like Poll Everywhere®) so that participants feel comfortable asking questions.
- Create alternate versions of the Activity 1 worksheet that focus on different settings, disciplines and professions that will be relevant to your audience.

# References

1. Young JQ, Van Merrienboer J, Durning S, Ten Cate O. Cognitive Load Theory: implications for medical education: AMEE Guide No. 86. *Med Teach.* 2014;36(5):371-384.
2. Sewell JL, Maggio LA, Ten Cate O, van Gog T, Young JQ, O'Sullivan PS. Cognitive load theory for training health professionals in the workplace: A BEME review of studies among diverse professions: BEME Guide No. 53. *Med Teach.* 2019;41(3):256-270.
3. Sweller J. Cognitive load during problem solving: effects on learning. *Cogn Sci.* 1988;12:257-285.
4. Schumacher DJ, Englander R, Carraccio C. Developing the Master Learner: Applying Learning Theory to the Learner, the Teacher, and the Learning Environment. *Acad Med.* 2013;88(11):1635-1645.
5. Naismith LM, Cavalcanti RB. Validity of Cognitive Load Measures in Simulation-Based Training: A Systematic Review. *Acad Med.* 2015;90(11 Suppl):S24-35.
6. White G. Mental load: helping clinical learners. *Clin Teach.* 2011;8(3):168-171.
7. Leppink J, Duvivier R. Twelve tips for medical curriculum design from a cognitive load theory perspective. *Med Teach.* 2016;38(7):669-674.
8. Vandewaetere M, Manhaeve D, Aertgeerts B, Clarebout G, Van Merrienboer JJ, Roex A. 4C/ID in medical education: How to design an educational program based on whole-task learning: AMEE Guide No. 93. *Med Teach.* 2015;37(1):4-20.
9. Fiorella L, Mayer RE. Eight Ways to Promote Generative Learning. *Educ Psychol Rev.*

2016;28(4):717-741.
