# Supplementary material for: Using Cognitive Load Theory to Improve Teaching in the Clinical Workplace
Source: MedEdPORTAL. 2020 Oct 2;16:10983. doi: 10.15766/mep_2374-8265.10983 (PMC7549387; doi:10.15766/mep_2374-8265.10983)
Supplement: Supplementary file 1 — Large-Group CLT Overview.pptxActivity 1 Small-Group Worked Example.docxActivity 2 Individual Activity Design.docxWorkshop Participant Evaluations.docxFollow-Up Survey.docxFacilitator Guide.docx [file mep_2374-8265.10983-s001.zip › _Educational_Summary_Report_10983.pdf]

MedEdPORTAL® The AAMC Journal of Teaching and Learning Resources

Original Publication

Open Access

Using Cognitive Load Theory to Improve Teaching in the Clinical Workplace

Manu V. Venkat, MD, Patricia S. O'Sullivan, EdD, John Q. Young, MD, MPP, PhD, Justin L. Sewell, MD, PhD, MPH*

*Corresponding Author: justin.sewell@ucsf.edu

Abstract

Introduction: Cognitive load theory (CLT) views working memory as the primary bottleneck for learning, as it is limited in both capacity and retention. CLT delineates three types of activities that impose on working memory: intrinsic load, germane load, and extraneous load. These three constructs have practical ramifications for direct teaching, learning environments, and curricular design. CLT could help educators across health professions improve quality of teaching, especially in demanding and unpredictable workplace environments. However, few educational resources exist to familiarize clinical workplace educators with CLT. **Methods:** We developed a 2-hour workshop focused on CLT's core concepts and practical applications, targeted at health professions' workplace educators. It featured large-group, small-group, and individual reflective activities. An end-of-workshop survey was administered, and a follow-up survey was sent to participants 2 months after the workshop. **Results:** A total of 134 educators attended the first two offerings of the workshop in two different states. Participants considered CLT as relevant to a variety of workplace teaching settings and activities. Participants' self-assessed familiarity with CLT on a 0-100 scale increased from a mean of 36 ($SD = 26$) before the workshop to 59 ($SD = 17$) after the workshop. At follow-up, participants scored an average of 85% on content knowledge questions. Approximately half of respondents to the follow-up survey stated they had made or planned to make specific changes to their workplace teaching leveraging tenets of CLT. **Discussion:** The workshop conveyed CLT concepts and primed participants to independently craft CLT-based interventions for their own teaching practices.

Keywords
Cognitive Load Theory, Workplace Teaching, Internal Medicine, Clinical Teaching/Bedside Teaching

Educational Objectives

By the end of this activity, learners will be able to:

1. Describe the Atkinson-Shiffrin model of human memory and basic tenets of cognitive load theory.
2. Describe the three types of cognitive load—intrinsic, germane, and extraneous—and give workplace examples of each.
3. Suggest ways to match intrinsic load to learners' levels of experience in their own workplace teaching setting.
4. Suggest ways to promote optimal germane load among learners within their own workplace teaching setting.
5. Suggest ways to diminish or mitigate potential sources of extraneous load in their own workplace teaching setting.

Citation:
Venkat MV, O'Sullivan PS, Young JQ, Sewell JL. Using cognitive load theory to improve teaching in the clinical workplace. *MedEdPORTAL*. 2020;16:10983. https://doi.org/10.15766/mep_2374-8265.10983

Introduction

Cognitive load theory (CLT) is a learning theory relevant to a wide variety of educational settings in the health professions. The theory focuses on the limited capacity of working memory compared with the significantly greater bandwidth of sensory and long-term memory.[1] The theory outlines how factors intrinsic or extraneous to a given learning task vie for that bandwidth. CLT delineates three specific types of cognitive load: intrinsic load, germane load, and extraneous load.[2] Intrinsic load occurs as learners perform the essential steps of the learning task itself. Germane load occurs as learners create and modify cognitive schemas that are stored in long-term memory for later retrieval and use. Extraneous load occurs when learners use working memory to focus on anything unrelated to task completion or learning; examples include task design (e.g., lack of integration between visual and verbal information) and environment factors, both internal (e.g., negative emotions) and external (e.g., noise). Learning is optimized when intrinsic load is matched to learners' experience level and extraneous load is minimized; in the motivated learner, this will promote working memory space

Copyright © 2020 Venkat et al. This is an open-access publication distributed under the terms of the Creative Commons Attribution license.

for activities contributing to germane load and, therefore, to learning.[1]

Just as other learning theories have demonstrated positive impacts on clinical teaching,[3] CLT principles can inform practical strategies to optimize learning within health professions education (HPE) settings.[4,5] In clinical workplace teaching, extraneous load can be minimized by designing learning environments that minimize distractions, disruptions, and multitasking; optimizing usability of visual resources such as informational displays and computer interfaces; and properly orienting trainees to learning settings and tasks.[6] Additionally, self-regulatory or metacognitive approaches can help learners manage stress and negative emotions to reduce their contributions to extraneous load.[7] Intrinsic load can be optimized by designing curricula that adapt learning task difficulty to a learner's skill level and by providing tools for teachers to familiarize themselves with learners' prior experience and competence. Simulation can be used to simplify tasks or break them into component parts (i.e., part-task approach), which is particularly useful when teaching novice learners.[5] A scaffolded curriculum allows learners to repeat tasks with decreasing amounts of support as they gain competence; a practical example of this is the 4C/ID model.[8] Germane load can be promoted through teacher engagement, interactive questioning of learners, encouraging reflection, and improving learner concentration and metacognition.[9] In a systematic review of cognitive load in professional workplace settings, the few studies that tested interventions to optimize cognitive load tended to demonstrate benefit.[4]

HPE workplaces are uniquely challenging and complex learning settings. Whereas in the classroom learners are the major focus, in the workplace the needs of multiple stakeholders must be met and balanced. HPE workplaces tend to be fast paced, complex, and even chaotic, and present substantial cognitive load burdens that can overwhelm learners (including multiple task-related demands, variability and unpredictability of daily schedules, and prioritization of patient care responsibilities over teaching[4,10]). This puts workplace learners at high risk of cognitive overload, which negatively impacts learning and performance, yet optimizing the distribution of cognitive load in workplace settings is especially challenging. Limited periods of continuity between teachers and learners, as well as idiosyncratic patient presentations and assignments, can hinder efforts to titrate task complexity to individual learners' levels. Rotation-based schedules can make it difficult to schedule spaced practice and repetitive recall over time, both of which are important for promoting germane load. Greater familiarity with CLT and how it can help workplace instruction could potentially help teachers in the health professions address these challenges and deliver a better experience for their learners.

While CLT is clearly relevant to HPE workplace learning, the scope of how the theory has been applied to workplace teaching and learning has been narrowly focused, with few examples of practical findings that can be easily translated to actual workplace environments.[4] Existing resources have provided a general orientation to CLT as it pertains to HPE,[1] a comprehensive scholarly review of studies of CLT in workplace learning,[4] and detailed steps for applying CLT to HPE curricular design.[7] These resources focus primarily on scholarship and do not address the practical needs of most workplace teachers, who rely on targeted skills workshops to help in areas outside their scope of expertise. These in-the-trenches educators could benefit from a resource that is theory based but promotes practical application of CLT within diverse workplace teaching settings and that provides sufficient guidance to allow teachers to design workplace learning experiences that attend to the cognitive load of learners. We sought to address this gap by applying our prior knowledge, scholarly experience with CLT, and workplace teaching experience to provide practical CLT-related concepts and principles that workplace health professions educators can put to use.

We developed and implemented a workshop to introduce core CLT concepts and applications to health professions educators, with a focus on practical application in workplace teaching. Given the variability of settings within which health professions educators work, we avoided an overly prescriptive approach to how workshop participants should implement CLT-based improvements to their teaching. Instead, the workshop equipped participants with a basic understanding of the theory before encouraging them to consider their own strategies for applying the theory to their workplace teaching.

This workshop was designed to benefit any individual who teaches in a health professions workplace setting, for example (but not limited to), teaching surgical residents in an operating room, medical students in an emergency department, nurse practitioner students in a primary care clinic, or pharmacy residents in an inpatient pharmacy. The primary goal was to assist teachers in the health professions in improving their learners' experiences using tenets of CLT. Secondary goals included assessing the efficacy of the workshop, understanding how teachers would envision CLT as applying to their workplace teaching settings, and considering challenges and barriers they might face as they attempt to implement those changes.

Copyright © 2020 Venkat et al. This is an open-access publication distributed under the terms of the Creative Commons Attribution license.

Methods

The workshop was offered to two audiences: the Annual Education Showcase at the University of California, San Francisco (April 30, 2018), and the Texas Educator's Academies Collaborative for Health Professions Southeast Annual Conference (May 11, 2018). Conference participants were invited to join the workshop regardless of their profession or level of training. No prerequisite knowledge was required of participants in order to attend.

The workshop was scheduled for 2 hours. It featured a mixture of large-group didactics, small-group discussion, and individual reflective activities. Two facilitators were recommended. Facilitators needed to have preexisting familiarity with CLT.

Participants were directed to bring a smartphone, tablet, or laptop. They were informed that they would receive an invitation to complete a follow-up survey approximately 2 months following the workshop.

The workshop time line was as follows:

- 0-30 minutes: facilitator-provided an overview of CLT in large-group PowerPoint format (Appendix A).
- 30-50 minutes: small-group activity (activity 1) examining CLT design principles using a worked example (itself a CLT-derived technique) in the setting of gastrointestinal endoscopy teaching (Appendix B).
- 50-60 minutes: discussion of insights with the whole group.
- 60-80 minutes: individual activity (activity 2) completed on participants' personal electronic devices, in which participants designed an activity personally relevant to them to which they could apply CLT principles to enhance efficacy of teaching (Appendix C).
- 80-120 minutes: sharing results of individual activity, debriefing activity, and synthesizing takeaways from activity in small-group and later large-group formats.

A web-based form administered in Qualtrics was used to facilitate activity 2 (Appendix C). The form prompted participants to select a workplace teaching activity relevant to their own professional role or, if they were uncertain what setting to discuss, to choose from a short list of prespecified HPE workplace activities. They were then asked to design one individualized CLT-related strategy within each of four domains (curricular design, direct teaching, learning environment, and metacognition)[4] for their selected workplace teaching task. The form also collected information about participants' profession, gender, the level(s) of learners they worked with, and their preexisting familiarity with CLT. Participants rated their satisfaction with the activity by completing evaluation instruments provided by the hosting organizations (Appendix D).

Participants received a follow-up survey that was sent out to them 2 months after the workshop, also using Qualtrics (Appendix E). The survey began with questions to assess their retention of knowledge presented during the workshop. It then inquired how the information presented during the workshop had impacted their thinking about teaching and whether it had prompted them to make any concrete changes to their teaching activities or curricula. If participants stated that they had made changes, they were prompted to discuss barriers or challenges they had experienced in making those changes. If they had made no changes to their teaching activities or curricula, they were asked to explain why.

To facilitate future replication of the workshop, we created a facilitator guide (Appendix F).

Results

A total of 134 educators participated in the two workshop offerings. The most commonly represented profession was medicine, followed by nursing, education, and dentistry (Table). Seventy-eight percent of those who indicated their gender identified as female (Table). Participants were generally unfamiliar with CLT prior to the workshop (mean familiarity on 0-100 scale = 36.2, *SD* = 26.5).

Table. Participant Characteristics

	Descriptive Statistic	
**Characteristic**[a]	**No. (%)**	***M* (*SD*)**
Profession[b]		
Medicine	38 (58)	
Nursing	10 (15)	
Education	6 (9)	
Dentistry	3 (5)	
Speech/occupational/physical therapy	1 (2)	
Pharmacology	1 (2)	
Other	6 (9)	
Years of teaching experience[c]		11.6 (11.6)
Gender[d]		
Female	51 (78)	
Male	14 (22)	
Level of learners taught[e]		
Preprofessional students	15 (12)	
Professional students	46 (37)	
Postgraduate trainees	37 (30)	
Faculty	25 (20)	

[a]Not all participants answered all questions. The number of responses for each characteristic is noted.
[b]*N* = 65.
[c]*N* = 61.
[d]*N* = 65.
[e]*N* = 123.

Copyright © 2020 Venkat et al. This is an open-access publication distributed under the terms of the Creative Commons Attribution license.

When asked to suggest a personally relevant teaching workplace activity to consider through the lens of CLT, participants listed activities among three primary categories. Sixty-one (46%) listed cognitive activities (e.g., patient presentations, patient counseling, difficult patient conversations, and designing faculty development workshops), 35 (26%) listed procedural activities (e.g., endotracheal intubation, nerve block, vaginal delivery, informed consent, lumbar puncture, dental crown preparation, and dermatologic procedures), and 37 (28%) listed workflow- or systems-related activities (e.g., teaching faculty to provide feedback, antibiotic stewardship, patient handoffs, and team skills).

Workshop Activity

Participants suggested a wide variety of ways to use CLT to optimize teaching. Below are selected responses for each of the four prompt domains. The quoted participant's profession is indicated in brackets.

1. Curricular design:
    - "Create a pre-rotation questionnaire to determine level of knowledge and adjust teaching based on score." [medicine]
    - "Use several different types of simulations geared towards different levels of learners (i.e., simulations with more support and less complex situations for less experienced learners, and less support and more complex situations for more experienced learners)." [education]
    - "Create standardized procedures for performing lumbar punctures in order to decrease instructor variability." [medicine]
    - "We might want to design a less complex [simulation] scenario for the early learners in this course. Have more complex scenarios for future courses that include higher-level learners." [medicine]
    - "Have the procedural skills sessions scattered over the course of intern year. Then have strategic on-site refresher simulation with senior residents over following 2 years." [medicine]
    - "Start with a short session followed by longer ones. Allow teams to decide on duration of longer ones. If fatigue sets in they should be able to break and come back, or schedule another one. Ask participants to report back on whether they thought each session was too short/long or just right." [other]
2. Direct teaching:
    - "Teachers should remain with learners when they start to use the new tool during surgery. They should ask pertinent questions and give guidance as necessary. Teachers should also meet with students after using the tool to reflect on what they learned, key [takeaways] and next steps to continue learning." [education]
    - "Empower learners to redirect the attending if the conversation becomes tangential." [medicine]
    - "Debrief after crisis situations [or] difficult patient encounters." [medicine]
    - "The surgeon should monitor the learner's ability to absorb the information regarding the introduction of the new equipment." [unspecified]
    - "Have a prebrief to let learner know you will be in there to support them. Have a safe word or phrase the learner can use to get assistance." [medicine]
    - "Anticipating heightened emotions in any resuscitation or simulation thereof, maintaining calm demeanor, stating that the newborn is tolerating their intubation attempt, or reminding them of option to 'tap out' if they (or supervisor) are uncomfortable." [medicine]
3. Learning environment:
    - "Teach in ICU first where [teaching] can be uninterrupted as opposed to in the OR with more production pressure and need for other activities at same time." [unspecified]
    - "Ask surgeon to turn down music (or turn off music) if appropriate." [medicine]
    - "Do morning rounds in a more isolated location." [medicine]
    - "Faculty should announce to the room that induction and intubation is taking place. If necessary, faculty should ask for the noise level to be kept low." [medicine]
    - "Arranging lab environment such that when teaching one small group [the] entire lab group can attend, listen, and learn." [speech, occupational, or physical therapy]
    - "Encourage that handoffs need to occur in a quiet setting; this needs to be role-modeled by attending and residents to encourage learning by the students that this type of environment is necessary." [education]
4. Metacognition:
    - "Review learning goals weekly, refine if they have been met or if they are proving too ambitious." [medicine]
    - "Have learners generate their own search strategy and questions to arrive at the piece of information that they need." [medicine]
    - "During the large group wrap-up asking students to quietly identify one personal germane learning goal that

Copyright © 2020 Venkat et al. This is an open-access publication distributed under the terms of the Creative Commons Attribution license.

they will carry forward into their own learning journeys based on today's sim/course." [medicine]

- "In simulation, challenge the learner with artificial distractions of alarms, frantic parents, etc. Afterwards, have them reflect on the impact, articulate a strategy for minimizing the distraction and run the case a second time so they can have a sense of efficacy." [medicine]
- "Ask them how they are feeling about expectations during feedback sessions." [medicine]
- "Have them think about what they would want to know for the handoff and how maybe someone with a different personality type might need different information." [medicine]

Participant Evaluations

Mean ratings of the two workshop offerings (all on a 5-point scale) ranged from 4.14 to 4.83 (Appendix D). Common positive comments were that the overall framework of CLT was relevant and that activity 2 (applying CLT concepts to participants' own workplace teaching settings) and subsequent discussion were beneficial. Common constructive feedback was that activity 1 (studying the worked example of colonoscopy teaching) was too complex and that its focus on the single procedural setting felt irrelevant to some participants.

Follow-up Survey

Out of 134 participants, 28 (21%) responded to the follow-up survey, of whom 23 (17%) completed questions assessing retention of content taught during the workshop (Appendix E, questions 1-6). Collectively, 85% of responses to these questions were correct, suggesting some retention of CLT concepts taught during the workshop. Mean self-perceived familiarity with CLT was 58.7 ($SD = 16.8$).

When prompted to discuss how information about CLT from the workshop had impacted their thinking about HPE teaching, many respondents cited examples of applications where understanding of CLT had proven helpful. These included clinical handoffs, faculty training, and simulation teaching. Some respondents reported not having spent any time thinking about CLT in the time since the workshop.

When asked if they had made, or planned to make, any specific changes to their workplace teaching or curricula as a result of learning about CLT, 11 respondents responded yes, and 12 responded no. Plans among those responding yes included moving teaching to a quieter setting, recognizing differences in optimal intrinsic load for different professionals involved in an interdisciplinary simulation exercise, and asking learners to evaluate new information in the context of their existing knowledge. The suggested changes were distributed between the goals of minimizing extraneous load, matching intrinsic load to learners' competency levels, and optimizing germane load. Challenges or barriers cited by those with plans for CLT-based interventions included low general awareness about CLT and inability to control extraneous load in workplace settings.

Among those who stated they were not making any change to their teaching based on takeaways from the CLT workshop, some respondents stated they lacked adequate time to do so. Others said that they worked in larger team settings in which changing curricula would be challenging. Some noted they were still digesting the workshop content or were unsure how to put CLT into practice despite their understanding of CLT in theory.

Discussion

We developed a workshop to address the lack of educational resources regarding CLT and its application to workplace teaching in the health professions. The workshop's design was predicated on two primary assumptions: (1) that CLT would be relevant across the range of workplace teaching applications in the health professions and (2) that once educators were equipped with a basic knowledge of the theory, they would be able to design and implement their own plan for using CLT principles to redesign their teaching practice.

This workshop-based CLT teaching resource directed towards workplace educators in the health professions filled a gap that we had recognized. A prior *MedEdPORTAL* resource regarding cardiac pacemaker placement utilizes concepts related to CLT, but it does not specifically teach CLT as a tool for addressing workplace teaching challenges.[11] We gave our workshop successfully at separate conferences in two different states and received similarly positive evaluations, suggesting that the workshop's value was not limited to one locality. The required instructor-to-participant ratio was low. The focus on practical application of CLT expanded the workshop's relevance to cover nearly anyone involved in HPE workplace teaching (potentially with some modifications, as discussed below) and could be expanded further to HPE workplace learners. In enrolling a broad group of participants, the workshop facilitated dialogue amongst teachers across different health professions. In our experience, the design of the workshop facilitated active learner engagement.

Based on the CLT knowledge questions in the follow-up survey, the workshop conveyed core CLT concepts with knowledge retention in the weeks to months following the workshop. Subjectively, respondents collectively indicated greater familiarity

Copyright © 2020 Venkat et al. This is an open-access publication distributed under the terms of the Creative Commons Attribution license.

with CLT at the postworkshop follow-up point than prior to the workshop. Longer-term knowledge retention would be useful to assess in the future but was not assessed in this study. Possible responder bias could exist; those who chose to respond may have been those who were more interested in CLT and who might have retained more information from the workshop.

One of this intervention's goals was assessing whether educators, once given a foundational understanding of CLT, would be able to independently craft plans for improving their own teaching practices based on the tenets of CLT. About half of respondents to the follow-up survey reported planning or implementing intentional changes in their teaching practices based on the workshop. Regrettably, multiple barriers to implementing CLT-based workplace teaching strategies were reported as well: inadequate time, inadequate buy-in from other faculty, and inadequate self-efficacy to effect change. These barriers provide important potential targets for future intervention.

The workshop and the participants' responses in the end-of-workshop activity have theoretical implications for the study of CLT in HPE. While numerous studies have leveraged CLT to study workplace settings,[4] they have been performed in a fairly restricted range of workplace settings. Workshop participants articulated multiple ways that CLT could apply across their diverse workplace teaching settings. They also suggested multiple specific teaching interventions, including procedural simulation sessions spaced at regular temporal intervals, teaching a skill in a setting with less time pressure before moving to practice in a setting with more time pressure, providing faculty with tools to address intense learner emotions, safewords that learners could use when cognitively overwhelmed, abstaining from playing music in the operating room when learners are present, allowing learners to decide on the duration of teaching sessions, and more rigorously modeling ideal behavior. These could inform future studies of CLT in HPE workplaces.

Limitations

Our results and conclusions must be tempered in light of methodological limitations. While we designed the workshop for diverse health care professions, participants were weighted heavily toward medicine (58%) and nursing (15%). We were not able to separate evaluation results by profession, and so, we do not know whether teachers across professions perceived similar benefits or not. Likewise, the specific medical procedural setting of colonoscopy in activity 1 might have felt irrelevant to participants who were not physicians or proceduralists (discussed further below).

The response rate to the follow-up survey was low (21%), likely because it was distributed 2 months after the workshop and included several short-answer questions that would take longer to complete than simple multiple-choice questions. This prevents us from making claims regarding the impact on the participant audience as a whole. However, there is evidence of benefit for the participants who chose to respond. The 85% accuracy rate for the CLT conceptual questions suggests conceptual learning and retention, and perceived familiarity with CLT increased to 58.7 from a baseline of 36.2 (on a 0-100 scale). Respondents' thoughtful responses regarding how they had used, or planned to use, CLT in their workplace teaching provide additional evidence suggesting learning.

Review of activity evaluations and the follow-up survey results revealed some weaknesses and areas for improvement. The workshop delivered a large amount of theoretical content during a single 2-hour session, which was not ideal for content retention. At the end of the workshop, some participants voiced uncertainty about the principles of CLT or how to apply them. To address this issue, a flipped classroom approach where basic information about CLT is delivered prior to the workshop could be used, or the workshop could be divided into several separate sessions. However, either of these changes would increase complexity and might decrease participation.

While activity 2 was generally well received, activity 1 prompted constructive criticism from several participants. We purposefully designed this activity as a worked example to reduce intrinsic load,[10] yet some participants evidently experienced increased intrinsic load, which they said was due to the complexity of the worksheet and the focus on colonoscopy (a specific procedural environment with which some participants had difficulty engaging). For future offerings of the workshop, we recommend offering procedural and cognitive worked example options that are representative of professions attending the workshop, as well as reducing the amount of text within the table. An interactive large-group approach might also promote more active learning during activity 1.

Among those who attempted to implement CLT-driven changes in their teaching practices, some mentioned low buy-in from other members of their teaching teams. This is not surprising given that CLT may not be familiar to many health professionals. To address this issue, future iterations of the workshop could target full teaching teams or units. Additionally, providing participants with a concise summary of CLT principles to give to a coworker—possibly in the form of a note card or brief animated

Copyright © 2020 Venkat et al. This is an open-access publication distributed under the terms of the Creative Commons Attribution license.

video—could reduce barriers to implementing team-wide CLT-driven interventions.

Reflective Critique

As education scholars, we desire to promote greater understanding of, engagement with, and practical integration of educational theory. We appreciate the particular relevance of CLT to workplace teaching in the health professions, and this workshop represents our efforts to disseminate this theory to workplace HPE teachers. Relevance of CLT is supported by the enthusiasm expressed by participants (appreciated by us during the workshop and evident in evaluation comments). At the same time, our analysis reveals a need to make the workshop more relevant to, and inclusive of, workplace teachers across professions and disciplines. For future offerings, we envision recruiting facilitators from other health professions, such as nursing, pharmacy, and dentistry, who can help develop revised materials that appeal to a diverse group of participants and who can connect on a professional level and promote greater participant engagement and strategies for buy-in. We also plan to leverage technology such as Poll Everywhere to promote more active learning during large-group sessions; this could be particularly useful during activity 1 and would be easily customizable for different professions and disciplines.

On a scholarly level, our prior work has found that study of CLT within workplace settings is narrowly focused.[4] We desire to broaden the scope of CLT study, including practical, translational studies. The suggestions for how CLT can be applied across professions and disciplines provide useful information to inform future research on CLT within HPE workplace settings.

Appendices

A. Large-Group CLT Overview.pptx

B. Activity 1 Small-Group Worked Example.docx

C. Activity 2 Individual Activity Design.docx

D. Workshop Participant Evaluations.docx

E. Follow-Up Survey.docx

F. Facilitator Guide.docx

All appendices are peer reviewed as integral parts of the Original Publication.

Manu V. Venkat, MD: Resident, Department of Medicine, New York-Presbyterian Hospital and Columbia University Medical Center

Patricia S. O'Sullivan, EdD: Professor, Department of Medicine, University of California, San Francisco School of Medcine; ORCID: https://orcid.org/0000-0002-8706-4095

John Q. Young, MD, MPP, PhD: Professor and Vice Chair for Education, Department of Psychiatry, Donald and Barbara Zucker School of Medicine at Hofstra/Northwell; ORCID: https://orcid.org/0000-0003-2219-5657

Justin L. Sewell, MD, PhD, MPH: Associate Professor, Department of Medicine and Division of Gastroenterology, Zuckerberg San Francisco General Hospital and University of California, San Francisco, School of Medicine; ORCID: https://orcid.org/0000-0003-4049-2874

Disclosures

None to report.

Funding/Support

None to report.

Ethical Approval

The University of California, San Francisco, Committee on Human Research approved this study.

References

1. Young JQ, Van Merrienboer J, Durning S, Ten Cate O. Cognitive load theory: implications for medical education: AMEE Guide no. 86. *Med Teach*. 2014;36(5):371-384. https://doi.org/10.3109/0142159X.2014.889290
2. Sweller J. Cognitive load during problem solving: effects on learning. *Cogn Sci*. 1988;12(2):257-285. https://doi.org/10.1207/s15516709cog1202_4
3. Schumacher DJ, Englander R, Carraccio C. Developing the master learner: applying learning theory to the learner, the teacher, and the learning environment. *Acad Med*. 2013;88(11): 1635-1645. https://doi.org/10.1097/ACM.0b013e3182a6e8f8
4. Sewell JL, Maggio LA, ten Cate O, van Gog T, Young JQ, O'Sullivan PS. Cognitive load theory for training health professionals in the workplace: a BEME review of studies among diverse professions: BEME Guide no. 53. *Med Teach*. 2019;41(3): 256-270. https://doi.org/10.1080/0142159X.2018.1505034
5. Naismith LM, Cavalcanti RB. Validity of cognitive load measures in simulation-based training: a systematic review. *Acad Med*. 2015;90(11)(suppl):S24-S35. https://doi.org/10.1097/ACM.0000000000000893
6. White G. Mental load: helping clinical learners. *Clin Teach*. 2011;8(3):168-171. https://doi.org/10.1111/j.1743-498X.2011.00458.x
7. Leppink J, Duvivier R. Twelve tips for medical curriculum design from a cognitive load theory perspective. *Med Teach*.

Copyright © 2020 Venkat et al. This is an open-access publication distributed under the terms of the Creative Commons Attribution license.

2016;38(7):669-674.
https://doi.org/10.3109/0142159X.2015.1132829

8. Vandewaetere M, Manhaeve D, Aertgeerts B, Clarebout G, Van Merriënboer JJG, Roex A. 4C/ID in medical education: how to design an educational program based on whole-task learning: AMEE Guide no. 93. *Med Teach*. 2015;37(1):4-20. https://doi.org/10.3109/0142159X.2014.928407

9. Fiorella L, Mayer RE. Eight ways to promote generative learning. *Educ Psychol Rev*. 2016;28(4):717-741. https://doi.org/10.1007/s10648-015-9348-9

10. Van Merriënboer JJG, Sweller J. Cognitive load theory in health professional education: design principles and strategies. *Med Educ*. 2010;44(1):85-93. https://doi.org/10.1111/j.1365-2923.2009.03498.x

11. Cousar J, Bohanske M, Hill J. Transvenous cardiac pacemaker educational resource. *MedEdPORTAL*. 2015;11:10107. https://doi.org/10.15766/mep_2374-8265.10107

Received: November 10, 2019
Accepted: April 18, 2020
Published: October 2, 2020

Copyright © 2020 Venkat et al. This is an open-access publication distributed under the terms of the Creative Commons Attribution license.
